# Supplementary material for: Near real-time surveillance of the SARS-CoV-2 epidemic with incomplete data
Source: PLoS Comput Biol. 2022 Mar 31;18(3):e1009964. doi: 10.1371/journal.pcbi.1009964 (PMC9004750; doi:10.1371/journal.pcbi.1009964)
Supplement: S1 Text — (PDF) [file pcbi.1009964.s001.pdf]

S1 Text

Additional information on model specification

Imputation model

For our imputation step, we first assume that missing DOS depends on the reporting date but occurs at random with respect to symptom onset date. Further, we assume that the distribution of the reporting delays conditional on DOR (Y), which is the sum of both the observed (Y^o) and the missing reporting delays (Y^m) can be parametrically approximated over time (t) and location (i) as a negative binomial (NB) distribution with mean $\mu_{i,t}$ and dispersion parameter $\theta_{i,t}$:

$$Y_{i,t} \sim NB(\mu_{i,t}, \theta_{i,t}) \qquad \text{eq. 1}$$

Under this assumption, the distribution of the missing delays over time and location $Y_{i,t}^m$ can be imputed from the parametric approximation of the distribution of the observed delays $Y_{i,t}^o$ over the same time and location.

We further assume that for a short period of time (τ) before the latest date of imputation d, which comprises consecutive reporting dates with lags $0,1,..u$, such as $\tau = d - \mathrm{u}, \mathrm{d} - u - 1,..d$, the reporting delay distribution conditional to DOR for the Spanish dataset remains relatively homogeneous. However, aiming to account for the weekly reporting patterns commonly present in infectious diseases reporting (e.g. fewer reporting during weekends), we also assume that the expectation of the one-week backward reporting delay distribution is affected by the day of the week. We model this dependency by using a categorical predictor w for the report day being a weekday (Monday to Friday) or a weekend (Saturday or Sunday). For each reporting day d, we first fit a generalized linear model to the observed reporting delay distribution over τ, using the log-link function in eq 1, with the reporting delay distribution modeled as a log-linear equation with expectation for day:

$$\log(\mu_{i,d}^o) \sim \beta\, w_{i,d} \qquad \text{eq. 2}$$

We then estimate for each day and location $\hat{\mu}_{i,d}^o$ and $\hat{\theta}_{i,d}^o$ using maximum likelihood [9]. We also estimate the standard error of the mean of both parameters, $SEM_{\hat{\mu}_{i,d}^o}$ and $SEM_{\hat{\theta}_{i,d}^o}$.

Using observations over τ, instead of only using observations at d allows us to increase the size of the observed delays at each location for the fitting step, as it becomes a particular limitation for *NB* regression when only a low number of observed delays (e.g. less than 300) is used, while still adjusting to weekday dependence and week-to-week changes on the reporting delays. Formulating alternative models for weekday dependence in the regression (or including other predictors such as age) can increase the accuracy of the imputation [1]. However, this will likely require further understanding of the reporting process and assumptions that need to be evaluated.

At each reporting day and location there are $m_{i,d}$ individuals with missing DOS. Thus, for each reporting day and location we generate a set of $n = m_{i,d}$random samples of missing backward reporting delays by predicting the reporting delays using the model in eq. 1 and eq. 2 and allowing for additional dispersion for the parameters $\mu_{i,d}^{m}$ and $\theta_{i,d}^{m}$, aiming to reliably estimate the uncertainty around the epidemic trends. Specifically, we model each parameter as having a normal distribution with mean equaling $\hat{\mu}$ and dispersion equaling $\hat{\theta}$ computed previously from the observed delays distribution, and the standard deviation equaling the computed *SEM* of each estimated parameter from observations.

$$\mu_{i,d}^{m} \sim Normal(mean = \ \hat{\mu}_{i,d}^{o} \ , sd = SEM_{\hat{\mu}_{i,d}^{o}} \ ) \qquad \text{eq 3.}$$

$$\theta_{i,d}^{m} \sim Normal(\text{mean} = \hat{\theta}_{i,d}^{o} \ , sd = SEM_{\hat{\theta}_{i,d}^{o}})$$

In our main model, we used a maximum lag u of 2 days (i.e., a 3-days period) to impute the missing delay samples, when the number of available observations for that period was 50 or more; when the number of observations in the previous period was less than 30, we increased the number of observations for the regression by increasing the maximum lag to 6 days (i.e., a 7-days period) and to 9 days (i.e, a 10-days period) if pooling from the previous again did not provide at least 30 observations. If the number of available observations after this procedure was smaller than 30 (such as the beginning of the outbreak for Murcia), we imputed the missing delays by subtracting the mean delay computed from the observed cases from the DOR of each case with missing DOS. We resampled 100 times to generate 100 time series of case-counts with complete DOS-DOR for each day and region. The sum of the observed and imputed cases became the total cases used for nowcasting.

Nowcasting model

We used *NobBS* to nowcast the number of yet unreported cases with DOS at day *d* that would be eventually reported under the assumption that the forward-delay conditional on DOS occurs as a negative binomial process. *NobBS* uses historical information on the reporting delay to predict the number of not-yet-reported cases in the present using a log-linear model of the number of cases. The implementation of *NobBS* requires the specification of 1) a sliding window for the time-varying reporting delay, 2) a maximum delay allowed for the window (L). We set up the moving window as the 75% of the total number of days in each of the periods of analysis and the maximum delay as the moving window-1 day.

The model underlying *NobBS* assumes that the observed number of cases at location i and day $d = 1, \dots D$ with delay $l = 0, \dots L$ follows a negative binomial distribution with parameters $p_{d,l}$ (probability of failure) and r (number of failures before stopping), such as:

$$n_{i,d,l} \sim NB(r, p_{i,d,l}) \quad \text{eq. 4}$$

$$p_{i,d,l} = r/(r + \lambda_{i,d,l}) \quad \text{eq. 5}$$

where $\lambda_{d,l}$ is the mean which is modeled as a log-linear equation depending on α_d which is the true signal and β_l, the probability of delay l, adapted from Verrall RJ et al [2]:

$$log(\lambda_{i,d,l}) = \alpha_{i,d} + log(\beta_{i,l}) \qquad \text{eq. 6}$$

As prior distributions on r,α_d,β_l we used the weakly informative priors used by *NobBS* by default: r is a Gamma distribution with parameters shape and rate$Gamma\ (60{,}20)$ to reflect moderate deviation from a Poisson distribution; $\alpha_d$is a first-order geometric random walk, aiming to capture the temporal autocorrelation of infections:

$$\alpha_{i,1} = N(0{,}0.001) \quad \text{eq. 7}$$

$$\alpha_{i,d>1} \sim N(\alpha_{i,d-1}, \tau_\alpha^2)$$

with parameter τ_α^2 modeled with the prior $\tau_\alpha^2 \sim Gamma\ (0.001{,}0.01)$; last, $\beta_{i,l}$,which a probability vector of length L (maximum delay), is a Dirichlet distribution where:

$$\beta_{i,l} \sim Dir(\theta) \quad \text{eq. 8}$$

$$\theta_{i,l} = \theta_{i,l}, \dots \theta_{i,L}$$

Note that the parametrization of the negative binomial distribution here is different from that of the imputation model in the previous section. The *NobBS* method can be implemented in R using the *NobBS* package (v1.2), which compiles in *JAGS* using the *rjags* package (v4.10).

Specifications of the generation interval distribution

We estimated the time-varying reproduction number $R_{t,\tau}$, where $\tau$is the window over which it is computed, using two approaches implemented in the R package *epiEstim* (v2.2.1): Wallinga and Teunis [3,4]) (WT) and Cori et al. (C) [3,4]. The package allows reconstruction of the reproductive number time series by using the epidemiological curve and a parametric approximation of the generation time (the time between a primary and a secondary case infection) with uncertainty in its distribution. The distribution is assumed to be Gamma, where the mean μ_{si} and standard deviation σ_{si} are also allowed to vary following a truncated normal distribution. To parameterize the Cori et al model in the main analysis, we used the median and standard deviation estimates from [20] and allowed for additional uncertainty: average μ_{gi} of 5 days (sd 1, min 2, max 7) and with an average σ_{gi} of 1.9 (sd 0.5, min 1, max 3). In sensitivity analyses, we used estimates of the mean and sd of the serial interval from [20] , assuming an average $\mu_{gi}$of 7.5 days (*sd* 2, min 4, max 11) and an average σ_{gi} of 3.4 (sd 1, min 1, max 7). However, the current WT estimate procedure in *epiEstim* does not allow the additional uncertainty around $\mu_{si}$and σ_{si}, thus we only used single values. Small-to-moderate deviations from the proposed parameterization did not change the trends of the time series, the time to become <1 and the uncertainty of the $R_t$estimates for each of the distributions.

We set up τ to one day as all epidemic curves included a sufficient number of incident cases at all time points for accurate estimates. We computed R_t from the 10,000 *NobBS* samples to produce the mean and 95% credibility interval of the R_t . For computational ease, we used a random sample of size 100 from the 10,000 samples since results did not materially change with a greater sample.

References:

1. Günther F, Bender A, Katz K, Küchenhoff H, Höhle M. Nowcasting the COVID-19 pandemic in Bavaria. Biom J. 2020. doi:10.1002/bimj.202000112

2. Verrall, R. J. (1994). Statistical methods for the chain-ladder technique. In *Casualty Actuarial Society Forum* (Vol. 1, pp. 393-446).

3. Wallinga J, Teunis P. Different epidemic curves for severe acute respiratory syndrome reveal similar impacts of control measures. Am J Epidemiol. 2004;160: 509–516.

4. Cori A, Ferguson NM, Fraser C, Cauchemez S. A new framework and software to estimate time-varying reproduction numbers during epidemics. Am J Epidemiol. 2013;178: 1505–1512.
