# Supplementary material for: Near real-time surveillance of the SARS-CoV-2 epidemic with incomplete data
Source: PLoS Comput Biol. 2022 Mar 31;18(3):e1009964. doi: 10.1371/journal.pcbi.1009964 (PMC9004750; doi:10.1371/journal.pcbi.1009964)
Supplement: S2 Text — (PDF) [file pcbi.1009964.s002.pdf]

S2 Text

Sensitivity of the imputation step

To evaluate the imputation step, we examined the symptoms-to-report delay distribution conditional on report day over time. The delay distribution changed over time, mostly showing continuously increasing dispersion over time and a tendency towards longer delays in the two regions. Differences between consecutive close days remained small but became more significant when using longer lags for comparison (Fig S1).

We further evaluated the accuracy of the imputation model producing 100 epidemic curves after randomly masking 10% and 40% of the cases with available DOS for the later stage and reconstructing the observed curve. As seen when comparing Fig S2 A and F vs B and G, masking increases the uncertainty by widening the range of the possible imputed time series. However, when a high proportion of cases are censored, the imputation model smooths the epidemic curve and reduces its initial slopes and global maxima. The smoothing was greater when using stronger assumptions (see methods). The Mean Absolute Percentage Errors (MAPE) did not substantially differ for the main model vs the alternatives with stronger assumptions were 13.1%, 14.6% and 14.2% respectively for Murcia, and 11.4%, 12.2%, 11.2% for Madrid. Mean delay backshift after randomly masking 40% of the cases provided less accurate estimates, conditional on the noise found at the time series by date of report (Fig S2 E and J). We also have evaluated alternative distributions for the reporting delays, namely the Poisson distribution and the more flexible Conway-Maxwell-Poisson distribution, none of which improved the estimates.

Last, we evaluated how major deviations from the missing-at-random assumptions would impact the imputation step, by reconstructing the true observed curve after a) randomly masking 20% cases among those with delays shorter than the median delay, or b) randomly masking 20% cases among those with delays longer than the median. The reconstruction is a skewed version of the smoothing that appears under the missing-at-random hypothesis. We observed that the imputed estimates were biased in the opposite direction of the bias in the masked data: when “missing” (masked) cases were mostly among those with longer reporting delay, the imputed curves underestimated cases of the increasing trend and overestimated on the decreasing trend (Fig S3 A and C); when missing cases were among those with shorter delays, the increasing trend was overestimated and the decreasing underestimate (Fig S3 B and D).
