# Supplementary material for: Near real-time surveillance of the SARS-CoV-2 epidemic with incomplete data
Source: PLoS Comput Biol. 2022 Mar 31;18(3):e1009964. doi: 10.1371/journal.pcbi.1009964 (PMC9004750; doi:10.1371/journal.pcbi.1009964)
Supplement: S3 Text — (PDF) [file pcbi.1009964.s003.pdf]

S3 Text

Sensitivity of the nowcasting step

Nowcast performance in real time with increasing available data

As shown in Fig 2 in the main analysis, for the early period of analysis in Madrid, the nowcasting approach slightly overestimated the observed trend. To judge the performance of the nowcast methods in a narrower time scale, under the hypothesis that increasing availability of data would increase the accuracy, we aimed to reconstruct the epidemic curve at each day for a period including the latest dates of the first two periods: 25 March to 8 April. Because the analysis cannot be performed only using the data available in the first period (as it ends by March 27), we used the data available in the second period but only for the specific dates, March 27 to 30 and April 1 to 8. Note that this second dataset is more complete (relative to the final data available) than the first one for the same time point of analysis (March 27).

Using this second dataset, the reconstructed curve performed very similarly for Madrid and Murcia, as shown in Fig S4. However, we observed that when large changes in underlying daily DOR report rate on a specific date affect the model nowcast, especially when in addition a high proportion of cases are missing the DOS. This can be seen for example for Madrid on Saturdays-Sundays (March 29 or April 4-5), but is less clear for Murcia. This is likely to have implications when the nowcast estimates are used for decision making in real time, as argued in the Discussion section.

Nowcast estimates using alternative approaches to determine the sliding window

We tested the effect of using different strategies for the *NobBS* window parameter. We compared the strategy used in the main analysis: 1) using a fixed-value sliding window (we selected 28 days), to the alternative observation-driven approach where 2) we changed the length of the sliding window over time depending on the observed delays (we selected the length of the window to be either period comprising 75% or 99% of the observed delay distribution).

As expected, the length of the window of analysis changed for each region when using a dynamic approach, with shorter windows using the narrowest criteria (75%) and longer with the widest criteria (99%): for example, for the intermediate period of analysis, we found 20 days of difference in Madrid (26 vs 6) and 15 days in Murcia (20 vs 5, Fig S5). Changing the criteria for determining the window of analysis did not correct for the overestimation of the nowcast curved in the earliest period vs that estimated later in time. Nevertheless, nowcast trajectories were affected by the window of analysis as seen in Figs S5 and S6. The length of the window of analysis influenced the accuracy of prediction; as expected, if too short the procedure is unable to nowcast the curve behind the latest day of the window (B and F). A dynamical window including 99% of the backward reporting delay accurately predicted cases later in time (C and G) similar to the main analysis (Fig 2). Finally, imputing by mean delay backshifting of incomplete data led to a less reliable epidemic curve and nowcast estimates (D and H).
