# Supplementary material for: Near real-time surveillance of the SARS-CoV-2 epidemic with incomplete data
Source: PLoS Comput Biol. 2022 Mar 31;18(3):e1009964. doi: 10.1371/journal.pcbi.1009964 (PMC9004750; doi:10.1371/journal.pcbi.1009964)
Supplement: S4 Text — (PDF) [file pcbi.1009964.s004.pdf]

S4 text

Sensitivity of the R_t estimation step

We compared the main analysis R_t estimates using data available in the intermediate period of analysis to those using a longer generation interval with mean 7.5 (SD 3.4), approximated from the serial interval reported by Li et al [1] (Fig S4, rows B and E). Longer generation interval leads to higher initial R_t, and the difference was more significant when using the WT approach. Time to R_t <1 did not change significantly when using the WT method but as expected was delayed relative to the C method by ~2 days, the difference with the longer generation interval. Finally, we examined the R_t estimates obtained from observed cases by DOR using the original generation interval of mean 5 (Fig S4, C and F) and shifted by the mean reporting delay (8 days for both regions). Estimates were significantly noisier in their trajectory and more difficult to interpret than those constructed using DOS, especially with smaller case counts as shown in F. In particular, the estimated date when R_t becomes < 1 using cases by date of report and backshift occurred 2 days earlier in Madrid for the C approach, and 8 days and 4 days earlier in Murcia for WT and C approaches respectively.

Sensitivity of R_t when using only observed cases by DOS

To evaluate how performing imputation and nowcasting improved the R_t estimates, we computed R_t using only observed cases by DOS and compared to the nowcasted curves using the full procedure for the 3 periods of analysis. As seen in Fig S8, when a high proportion of cases are missed along the period of analysis and/or on a specific date, $R_t$is underestimated. For example, for the first period of analysis the estimated time point for R_t<1 using only available cases by DOS is earlier in time (Fig S8 A,D for Madrid and Murcia). As more information is available (i.e. latest period of analysis, Fig S8 C,F) the estimates using available DOS are less biased.

Sensitivity of R_t to changes on ascertainment rate

For our dataset we do not expect to have major changes in ascertainment rates because clinical and surveillance procedures, as well as capacities did not substantially change during the analyzed periods. However, to illustrate the impact of potential changes in ascertainment on the nowcasting and the subsequent estimation of R_t in our procedure, we evaluated how a major drop (a 50% lower ascertainment) occuring during the initial period of analysis would impact the estimates. This is a plausible change, especially for the first weeks of transmission of COVID-19. The overall shape and trends of the nowcast curves were affected when simulating a substantial change in ascertainment rate, as shown in Fig S5 A-C and G-I, but were only slightly affected with regards to the peak of transmission and consequent R_t estimation.

References:

1. Li Q, Guan X, Wu P, Wang X, Zhou L, Tong Y, et al. Early Transmission Dynamics in Wuhan, China, of Novel Coronavirus-Infected Pneumonia. N Engl J Med. 2020;382: 1199–1207.
