# Supplementary material for: Near real-time surveillance of the SARS-CoV-2 epidemic with incomplete data
Source: PLoS Comput Biol. 2022 Mar 31;18(3):e1009964. doi: 10.1371/journal.pcbi.1009964 (PMC9004750; doi:10.1371/journal.pcbi.1009964)

**Fig S1.** Empirical distribution and approximated functions of the reporting delay conditional on report date in the regions of Madrid and Murcia, Spain, March 1-April 16, 2020. The blue columns represent the true observed proportion. The grey line and ribbon represent the median and 95% uncertainty interval of the prediction under the fitted negative binomial distribution.

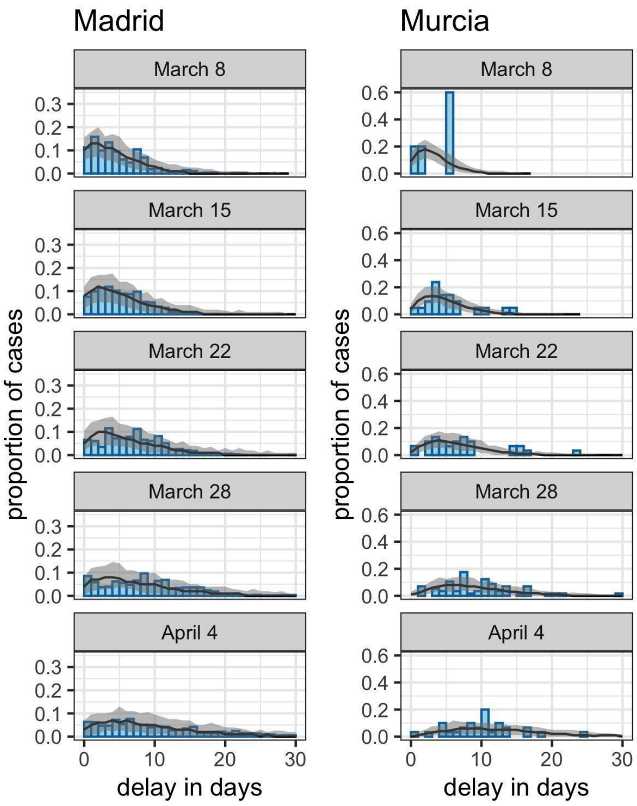

Supplement: S1 Fig — The blue columns represent the true observed proportion. The grey line and ribbon represent the median and 95% uncertainty interval of the prediction under the fitted negative binomial distribution. (PDF) [file pcbi.1009964.s005.pdf]
