# Supplementary material for: Near real-time surveillance of the SARS-CoV-2 epidemic with incomplete data
Source: PLoS Comput Biol. 2022 Mar 31;18(3):e1009964. doi: 10.1371/journal.pcbi.1009964 (PMC9004750; doi:10.1371/journal.pcbi.1009964)

Fig S2. Plotting the sum of imputed and observed epidemic curves (black line median, ribbon 95% CI) in the regions of Madrid and Murcia, Spain, March 1-April 16, 2020, estimated after A,F) randomly masking 10% of available reporting delays and using the main imputation approach, B,G) randomly masking 40% of available reporting delays and using the main imputation approach, C,H) randomly masking 40% of available reporting delays and estimating from a 7-day window of observations and allowing additional variance for the dispersion parameter D,I) randomly masking 40% of available reporting delays and using a single value for the mean in the negative binomial, and E,J) randomly masking 40% of available reporting delays and imputing by mean delay backshifting. Blue columns represent true observed case counts by day of symptoms onset.

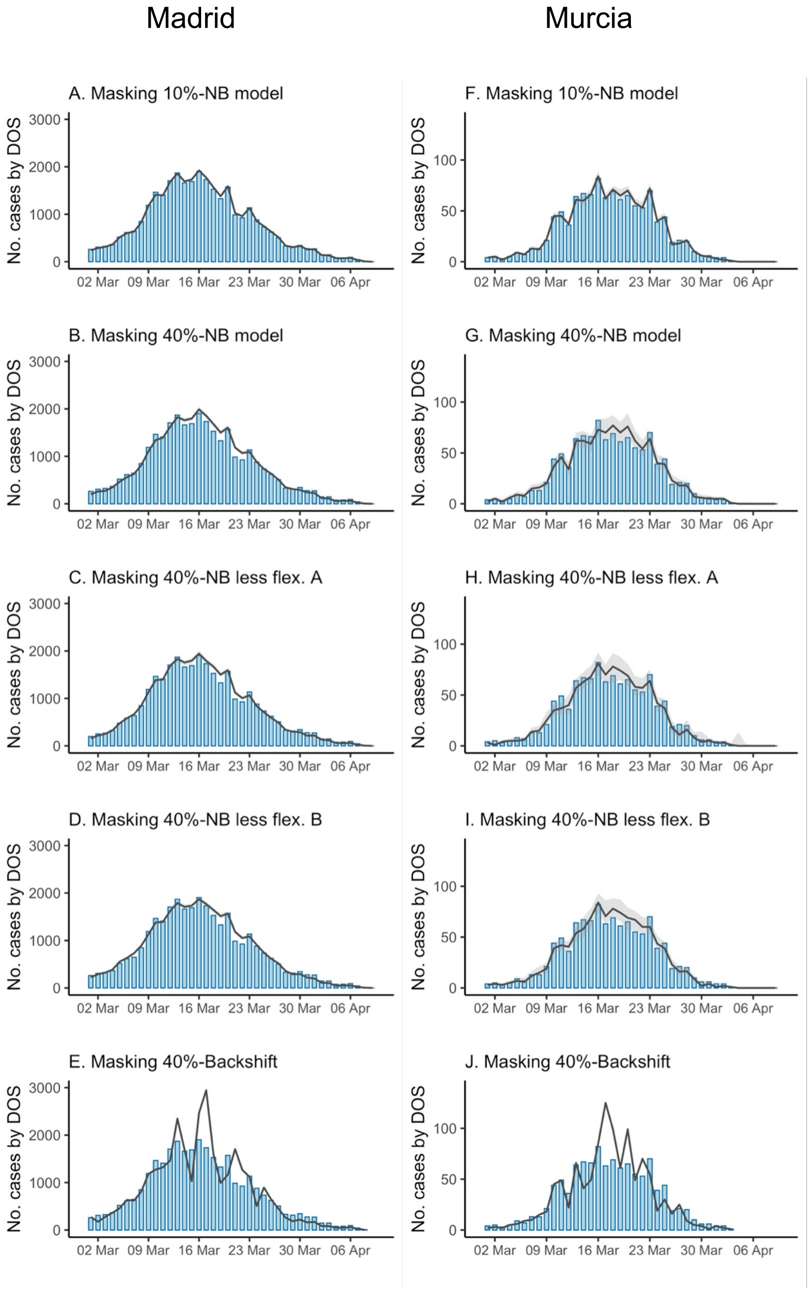

Supplement: S2 Fig — Plotting the sum of imputed and observed epidemic curves (black line median, ribbon 95% CI) in the regions of Madrid and Murcia, Spain, March 1-April 16, 2020, estimated after A,F) randomly masking 10% of available reporting delays and using the main imputation approach, B,G) randomly masking 40% of available reporting delays and using the main imputation approach, C,H) randomly masking 40% of available reporting delays and estimating from a 7-day window of observations and allowing additional variance for the dispersion parameter D,I) randomly masking 40% of available reporting delays and using a single value for the mean in the negative binomial, and E,J) randomly masking 40% of available reporting delays and imputing by mean delay backshifting. Blue columns represent true observed case counts by day of symptoms onset. (PDF) [file pcbi.1009964.s006.pdf]
