# Supplementary material for: Near real-time surveillance of the SARS-CoV-2 epidemic with incomplete data
Source: PLoS Comput Biol. 2022 Mar 31;18(3):e1009964. doi: 10.1371/journal.pcbi.1009964 (PMC9004750; doi:10.1371/journal.pcbi.1009964)

Fig S3. Plotting the sum of imputed and observed epidemic curves (black line median, ribbon 95% CI) in the regions of Madrid and Murcia, Spain, March 1-April 16, 2020, estimated after A, B) randomly masking 20% of available reporting delays among cases with delays longer than the median and C, D) randomly masking 20% of available reporting delays among cases with delays shorter than the median. Blue columns represent true observed case counts by day of symptom onset.

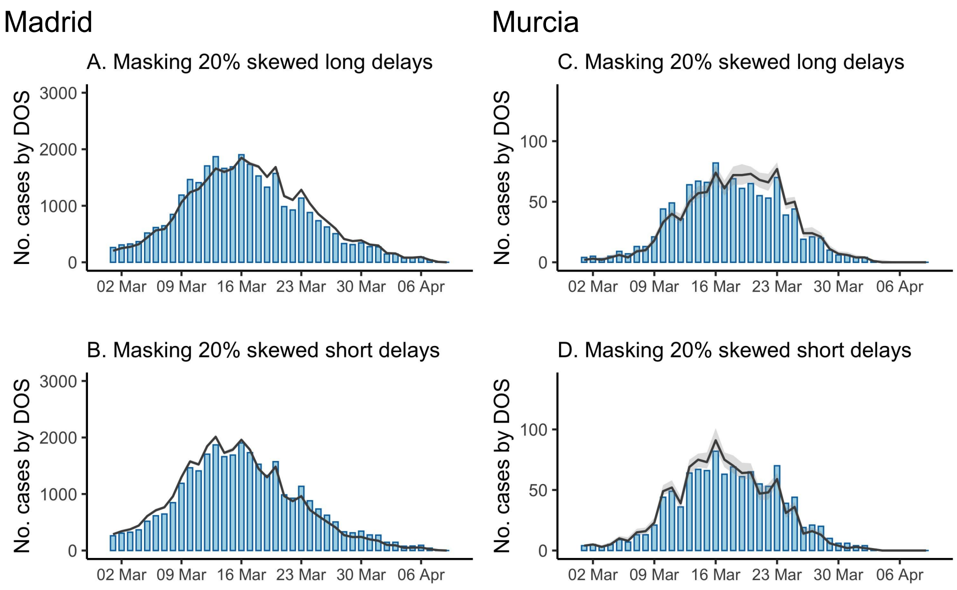

Supplement: S3 Fig — Plotting the sum of imputed and observed epidemic curves (black line median, ribbon 95% CI) in the regions of Madrid and Murcia, Spain, March 1-April 16, 2020, estimated after A, B) randomly masking 20% of available reporting delays among cases with delays longer than the median and C, D) randomly masking 20% of available reporting delays among cases with delays shorter than the median. Blue columns represent true observed case counts by day of symptom onset. (PDF) [file pcbi.1009964.s007.pdf]
