# Supplementary material for: Near real-time surveillance of the SARS-CoV-2 epidemic with incomplete data
Source: PLoS Comput Biol. 2022 Mar 31;18(3):e1009964. doi: 10.1371/journal.pcbi.1009964 (PMC9004750; doi:10.1371/journal.pcbi.1009964)
Supplement: S4 Fig — Nowcast estimates in Madrid and Murcia (orange lines are median estimates, ribbons span 2.5 and 97.5 percentiles, observed cases with known date of onset of symptoms for the late period of analysis (blue columns), cases with imputed date of onset of symptoms for the late period of analysis (grey columns) and nowcasted uncertainty range for the late period of analysis (grey ribbon); Mon: Monday, Tue: Tuesday, Wed. Wednesday, Thu.: Thursday, Fr.: Friday, Sat.: Saturday, Sun.: Sunday. (PDF) [file pcbi.1009964.s008.pdf]

Fig S4. Evaluation of the reconstructed epidemic curves at each day between March 25- April 8 using the data available during the intermediate period of analysis of the initial SARS-CoV-2 outbreak in the regions of Madrid and Murcia, Spain.

Madrid

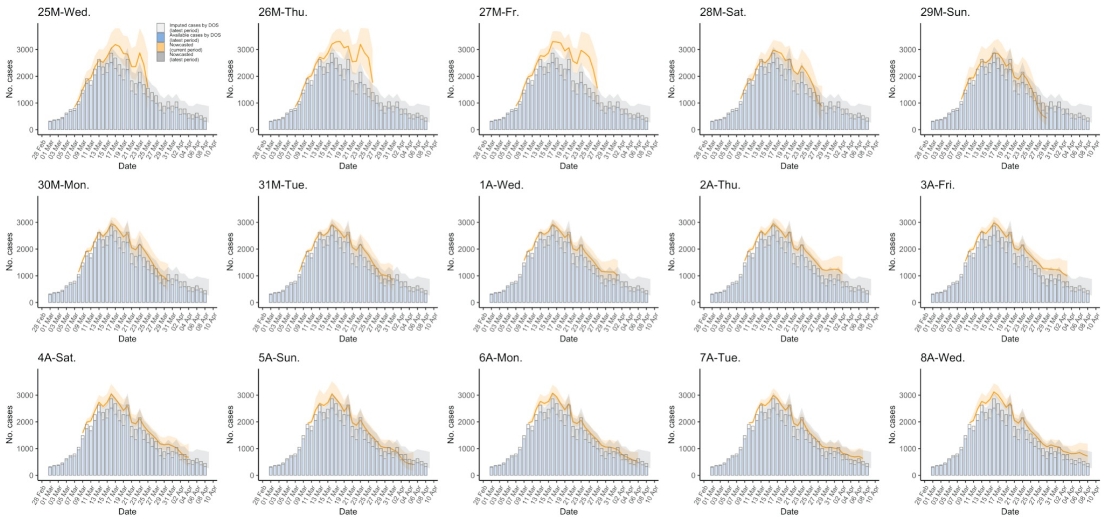

Murcia

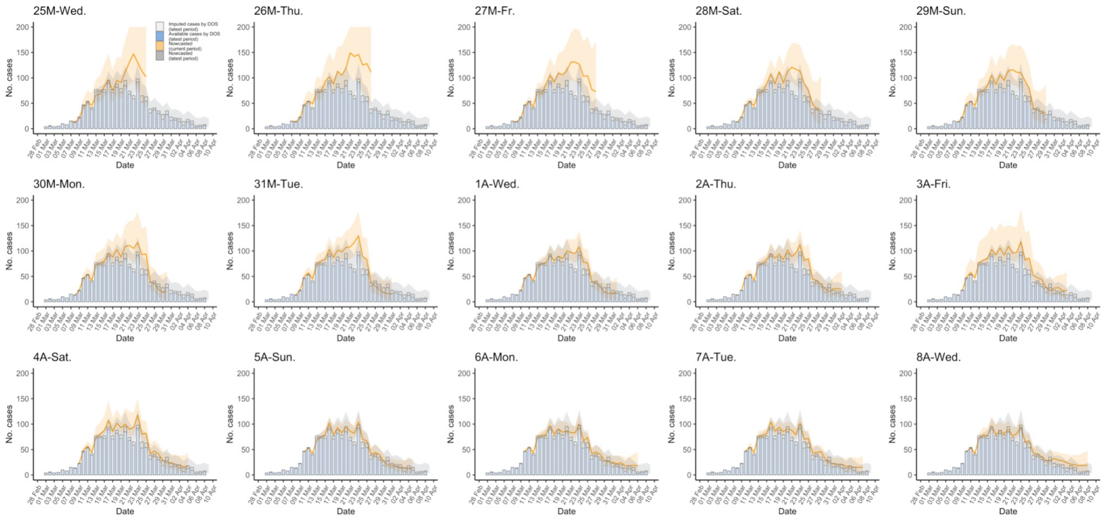

Nowcast estimates in Madrid and Murcia (orange lines are median estimates, ribbons span 2.5 and 97.5 percentiles, observed cases with known date of onset of symptoms for the late period of analysis (blue columns), cases with imputed date of onset of symptoms for the late period of analysis (grey columns) and nowcasted uncertainty range for the late period of analysis (grey ribbon); Mon: Monday, Tue: Tuesday, Wed. Wednesday, Thu.: Thursday, Fr. : Friday, Sat. : Saturday, Sun.: Sunday.
