# Supplementary material for: Near real-time surveillance of the SARS-CoV-2 epidemic with incomplete data
Source: PLoS Comput Biol. 2022 Mar 31;18(3):e1009964. doi: 10.1371/journal.pcbi.1009964 (PMC9004750; doi:10.1371/journal.pcbi.1009964)
Supplement: S6 Fig — Showing nowcast estimates in the intermediate period of analysis (yellow lines represent the median and ribbons span the 2.5 and 97.5 percentiles) 1) using a fixed window of 28 days 2) using a window that accounts for 75% of the delays available from the latest period of observations (B and F) 3) using a window that account for 99% of the observed delays (C and G); and 4) imputing through backshifting the report date by mean delay (D and H). Dashed lines and light grey ribbon represent nowcasted cases later in time using the main approach (late period of analysis). Faded blue columns represent observed cases by DOS, faded grey columns represent median imputed cases by DOS; dark grey ribbons (in A,B,C, D, E and F) represent observed plus imputed 2.5 and 97.5 percentiles. (PDF) [file pcbi.1009964.s010.pdf]

Fig S6. Epidemic curves estimated using alternative values for the *NobBS* sliding window and the data available during the intermediate analysis of the initial SARS-CoV-2 outbreak in the regions of Madrid and Murcia, Spain, March 1-April 9, 2020, comparison with backshifted epidemic curves by report date, and curves obtained in late period of analysis March 1-April 16 using the main approach.

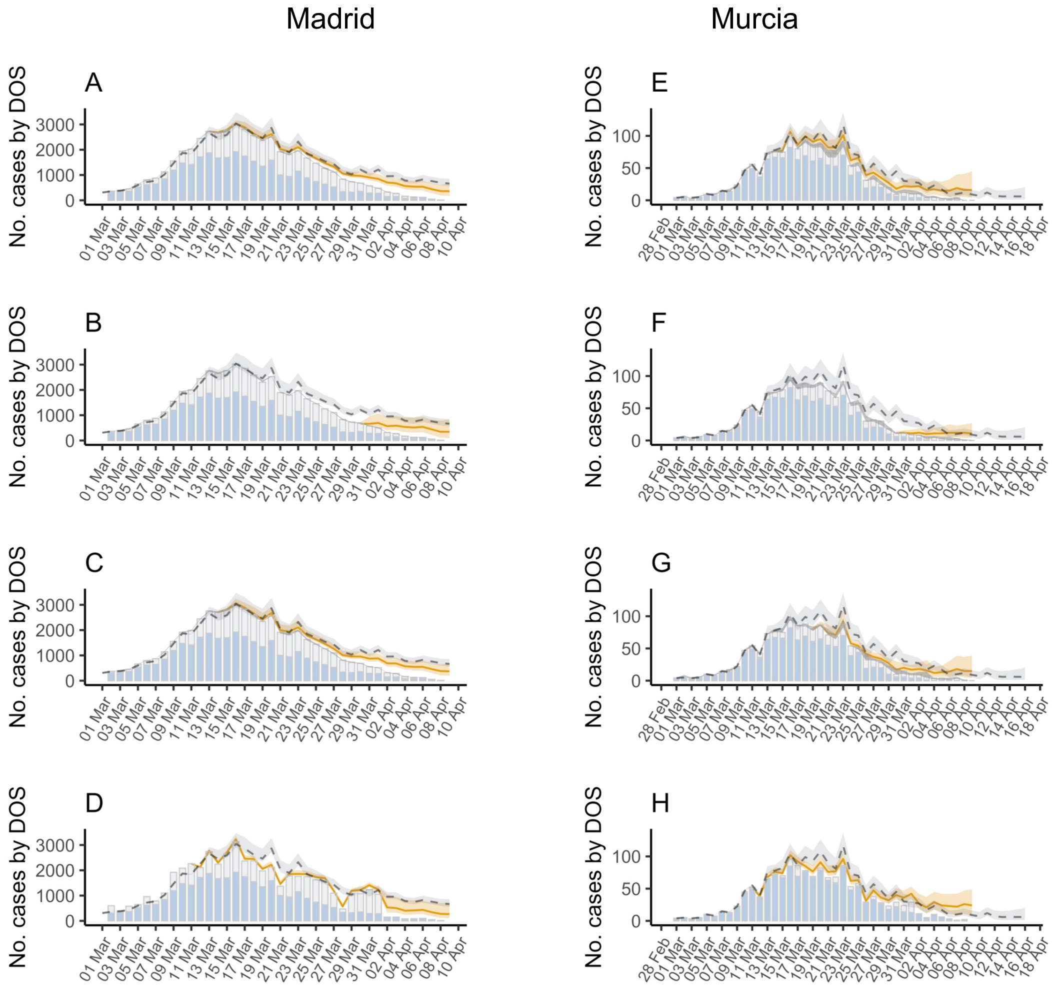

Showing nowcast estimates in the intermediate period of analysis (yellow lines represent the median and ribbons span the 2.5 and 97.5 percentiles) 1) using a fixed window of 28 days 2) using a window that accounts for 75% of the delays available from the latest period of observations (B and F) 3) using a window that account for 99% of the observed delays (C and G); and 4) imputing through backshifting the report date by mean delay (D and H). Dashed lines and light grey ribbon represent nowcasted cases later in time using the main approach (late period of analysis). Faded blue columns represent observed cases by DOS, faded grey columns represent median imputed cases by DOS; dark grey ribbons (in A,B,C, D, E and F) represent observed plus imputed 2.5 and 97.5 percentiles.
