# Supplementary material for: Near real-time surveillance of the SARS-CoV-2 epidemic with incomplete data
Source: PLoS Comput Biol. 2022 Mar 31;18(3):e1009964. doi: 10.1371/journal.pcbi.1009964 (PMC9004750; doi:10.1371/journal.pcbi.1009964)
Supplement: S7 Fig — Showing sensitivity analysis of the Rt estimates. Rows A and D plots Rt for WT (red) and C (purple) approaches using the nowcast estimates and a generation interval of mean 5 (1.9 SD). Estimates are also obtained using a longer generation interval of mean 7.5 (3.4 SD) shown in rows B and E; Rows C and F shows Rt estimates using the shorter serial interval but calculated from the observed cases by date of report. Vertical lines indicate the day when Rt <1 (red dashed line for WT, purple dashed line for C). (PDF) [file pcbi.1009964.s011.pdf]

Fig S7. Reproductive numbers estimated comparing 2 different generation intervals on nowcasted curves and curves by report date using the data available during the intermediate analysis of the initial SARS-CoV-2 outbreak in the regions of Madrid and Murcia, Spain, March 1-April 9, 2020

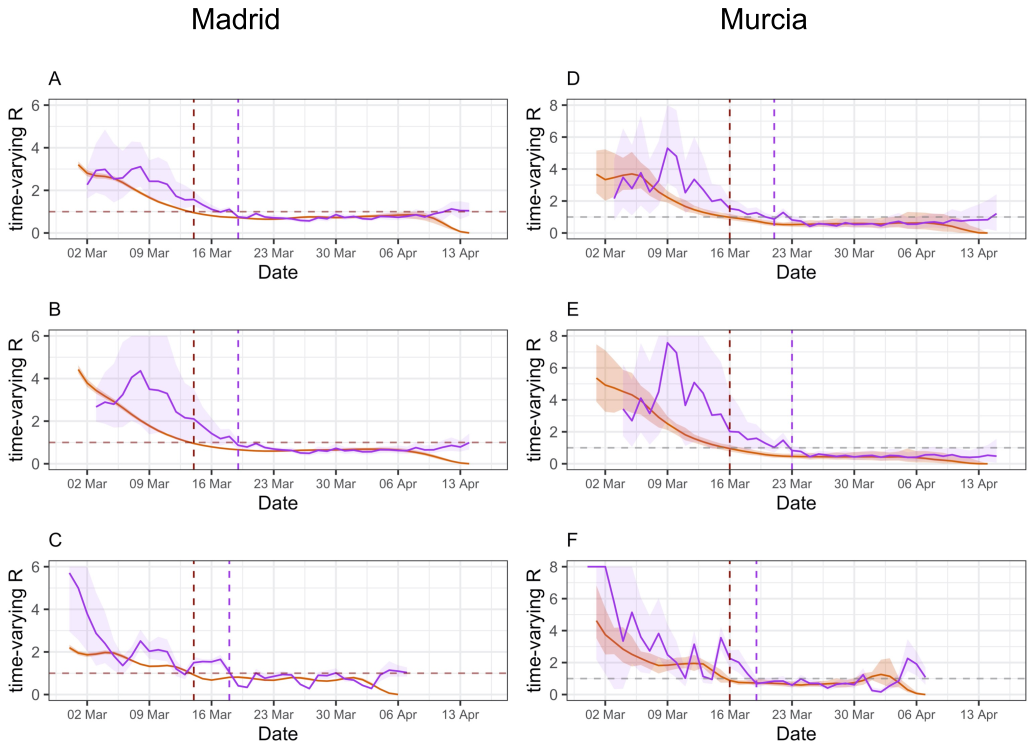

Showing sensitivity analysis of the R_t estimates. Rows A and D plots R_t for WT (red) and C (purple) approaches using the nowcast estimates and a generation interval of mean 5 (1.9 SD). Estimates are also obtained using a longer generation interval of mean 7.5 (3.4 SD) shown in rows B and E; Rows C and F shows R_t estimates using the shorter serial interval but calculated from the observed cases by date of report. Vertical lines indicate the day when R_t <1 (red dashed line for WT, purple dashed line for C).
