# Supplementary material for: Near real-time surveillance of the SARS-CoV-2 epidemic with incomplete data
Source: PLoS Comput Biol. 2022 Mar 31;18(3):e1009964. doi: 10.1371/journal.pcbi.1009964 (PMC9004750; doi:10.1371/journal.pcbi.1009964)
Supplement: S8 Fig — Lines are median estimates, ribbons span 2.5 and 97.5 percentiles. Vertical lines indicate the day when Rt <1 (Rt estimated from nowcasted curves are shown in red dashed line for WT, purple dashed line for C; Rt estimated from available cases by DOS are shown in blue). (PDF) [file pcbi.1009964.s012.pdf]

Fig S8. Reproductive numbers estimated using only available cases by DOS vs. nowcasted curves and curves by report date using the data available during the three periods of analysis the initial SARS-CoV-2 outbreak in the regions of Madrid and Murcia, Spain, March 1-April 9, 2020

Early analysis (March 1-27) | Intermediate analysis (March 1-April 9) | Late analysis (March 1-April 16)

Madrid

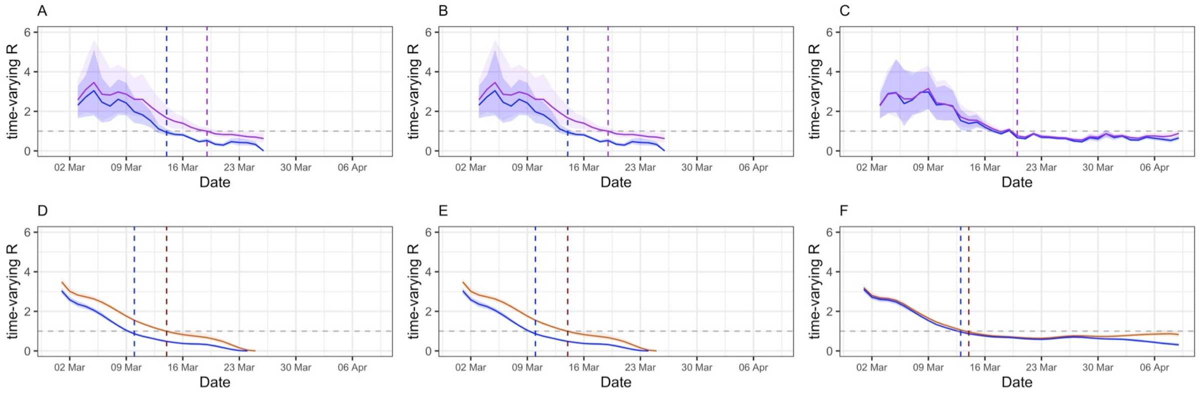

Murcia

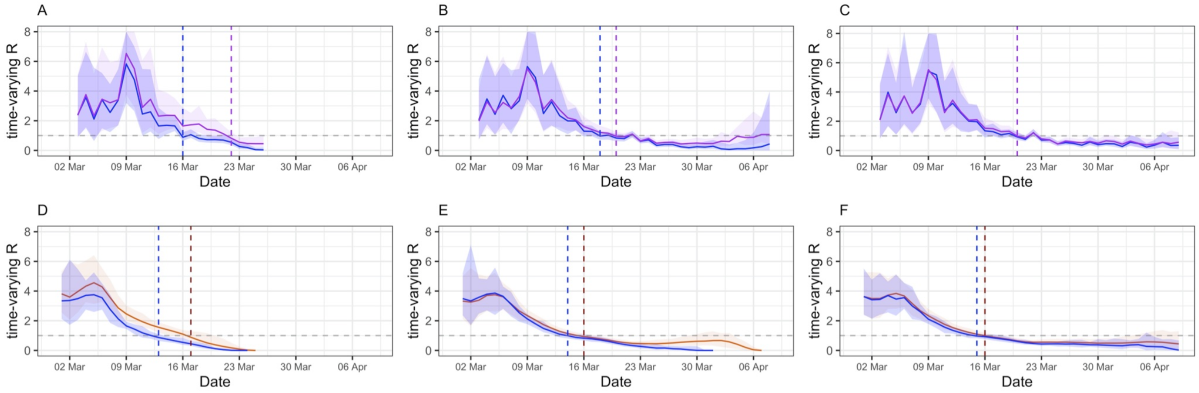

Lines are median estimates, ribbons span 2.5 and 97.5 percentiles. Vertical lines indicate the day when R_t <1 (R_t estimated from nowcasted curves are shown in red dashed line for WT, purple dashed line for C; R_t estimated from available cases by DOS are shown in blue)
