# Supplementary material for: Near real-time surveillance of the SARS-CoV-2 epidemic with incomplete data
Source: PLoS Comput Biol. 2022 Mar 31;18(3):e1009964. doi: 10.1371/journal.pcbi.1009964 (PMC9004750; doi:10.1371/journal.pcbi.1009964)
Supplement: S9 Fig — Epidemic curves and Rt estimates using the data available during the the intermediate analysis of the initial SARS-CoV-2 outbreak in the regions of Madrid and Murcia, Spain, March 1-April 9, 2020 (A,D and G,J) and comparison of the same estimate approach after randomly subtracting 50% of cases during the first 2 weeks (without including peak transmission, B,E and H,K) and during the first 4 weeks (including peak transmission, C,F and I,L). DOR: date of report; Lines are median estimates, ribbons span 2.5 and 97.5 percentiles. Vertical lines indicate the day when Rt <1 (red dashed line for WT, purple dashed line for C). (PDF) [file pcbi.1009964.s013.pdf]

Fig S9. Epidemic curves and R_t estimates using the data available during the the intermediate analysis of the initial SARS-CoV-2 outbreak in the regions of Madrid and Murcia, Spain, March 1-April 9, 2020 (A,D and G,J) and comparison of the same estimate approach after randomly subtracting 50% of cases during the first 2 weeks (without including peak transmission, B,E and H,K) and during the first 4 weeks (including peak transmission, C,F and I,L)

Madrid

Full ascertainment | 50% ascert., first 2 weeks | 50% ascert., first 4 weeks

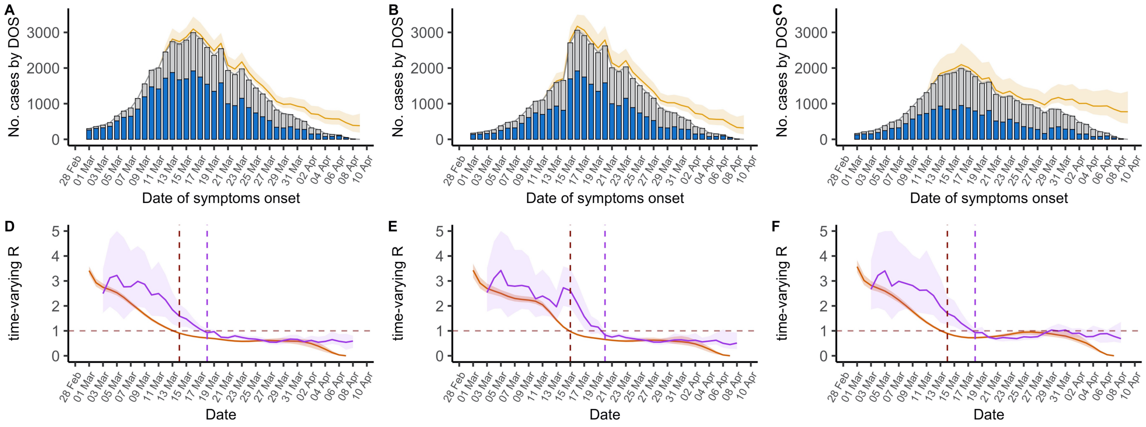

Murcia

Full ascertainment | 50% ascert., first 2 weeks | 50% ascert., first 4 weeks

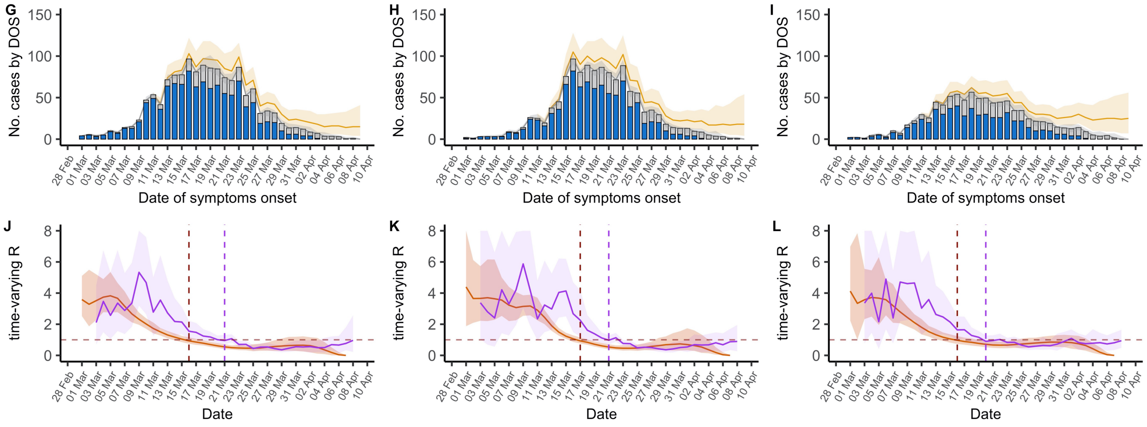

DOR: date of report; Lines are median estimates, ribbons span 2.5 and 97.5 percentiles. Vertical lines indicate the day when R_t <1 (red dashed line for WT, purple dashed line for C)
