# Supplementary material for: Near real-time surveillance of the SARS-CoV-2 epidemic with incomplete data
Source: PLoS Comput Biol. 2022 Mar 31;18(3):e1009964. doi: 10.1371/journal.pcbi.1009964 (PMC9004750; doi:10.1371/journal.pcbi.1009964)
Supplement: Attachment — Submitted filename: Response_To_reviewer.pdf [file pcbi.1009964.s016.pdf]

Dear editors Dr B M Althouse and Dr T Britton,

Thank you for allowing us to send a revised version of our manuscript for further review in *PLOS Computational Biology*.

We have carefully addressed all comments queried by Reviewer-2. This has mostly included a thorough review of the statistical terminology for clarity as well as editing for underscoring the potential solutions to address the limitations of the approach in further work.

Please find below our responses to the Reviewer-2 queries.

Sincerely, on behalf of all co-authors,

Pablo M De Salazar

Reviewer #2: I still have some major comments and remarks w.r.t to the current form of the manuscript that should be addressed:

Imputation model

Thanks for the clarifications with respect to the imputation model(s) in your last version of the manuscript. Based on these, I have some comments:

A) General description of Imputation model:

1. The formulation of the imputation model based on the formulas eq1-eq3 in S1 continues to be unclear, Eq 2 and Eq 3 seem to be erroneous. In a way, they imply that the parameters are supposed to follow an additional model (as e.g., in a Bayesian hierarchical model) but this seems not to be the case based on the written text. Furthermore, it does not make sense that mu_it follows a normal distribution with expectation mu_it (i.e., itself) and why lamda_i,t is supposed to follow a normal distribution with expectation mu_i,t. Also it does not make sense that the variance parameters/standard deviation is shared between mu_i,t and theta_it.

2. From what I understand from the written text the backward delay distribution for reporting day t, t=1,…T, is just estimated by ML and you estimate the expectation and overdispersion parameter of a negative binomial distribution, where the data correspond to the (observed) delays of all individuals with reporting date t-1, t, and t+1. In this case, the parameters do not have any distribution but are assumed to be fixed, and only their estimates hat(mu) and hat(theta) have an approximate distribution/associated standard error based on ML-theory.

3. You then perform the imputation by sampling parameters of a negative binomial distribution based on the (marginal) approximate normal distribution of the estimated parameters hat(mu) and hat(theta) and sampling reporting delays for each individual from the corresponding distribution. Please provide a valid description of your mathematical models!

We have now rewritten the imputation paragraph in the methods as well as section S1 of the supplementary to clarify the imputation model for addressing the previous points A1-A3.

In the Methods sections:

"We first assumed that missing DOS occurred at random with respect to symptom onset date, and that reporting delays conditional on DOR can be modeled over time t and location i as a negative binomial distribution with mean parameter $\mu_{i,t}$ and dispersion parameter $\theta_{i,t}$. Therefore, samples of the missing backward reporting delays $Y_{i,t}^m$ can be obtained from the parametric approximation of the observed backward reporting delays over the same time and location, $Y_{i,t}^o$, resulting in the following model:

$$Y_{i,t}^m \simeq Y_{i,t}^o$$

$$Y_{i,t}^o \sim NB(\mu_{i,t}^o, \theta_{i,t}^o)$$

We dynamically estimated the parametric delay distribution at each day by regressing the backward delay distribution from all cases with available DOS pooled over a period of time τ comprising all dates between the day of imputation d and a lag u (i.e., $\tau = d - u, \ldots d$) and using maximum likelihood [9]. Using observations over τ, instead of only using observations at d allows us to increase the size of the observed delays at each location for the fitting step. However, to adjust for variation on the reporting patterns due to day of the week (i.e., reduced reporting during weekends compared to weekdays) as well as different short-term dynamical trends (i.e. increasing/decreasing trend over a week), we modeled the mean delay conditional to the categorical predictor w being the reporting date weekday or weekend:

$$\mu_{i,\tau}^o \sim \beta\, w_{i,\tau}^o$$

For the main analysis we pooled from a 3 -days period when the number of available observations were 50 or more, and sequentially increased τ to 7 or 10 days if the total number of observations in τ were smaller than 30. When the number of observations included in the longest period of time (10 days) were less than 30, we simply imputed the missing delays by subtracting the observed mean delay from the DOR of each case with incomplete information. Last, for imputing the missing DOS at each reporting day d and aiming to reliably estimating the uncertainty around the epidemic trends, we sampled from a randomly generated negative binomial distribution where $\mu_{i,d}^m$ and $\theta_{i,d}^m$ were modeled as having both a normal distribution with the mean set to the estimated $\hat{\mu}_{i,d}^o$ and $\hat{\theta}_{i,d}^o$, and the standard deviation set to the standard error of the mean."

In the Supplementary:

"Section S1. Additional information on model specification

Imputation model

For our imputation step, we first assume that missing DOS depends on the reporting date but occurs at random with respect to symptom onset date. Further, we assume that the distribution of the reporting delays conditional on DOR (Y), which is the sum of both the observed (Y^o) and the missing reporting delays (Y^m) can be parametrically approximated over time (t) and location (i) as a negative binomial (NB) distribution with mean $\mu_{i,t}$ and dispersion parameter $\theta_{i,t}$:

$$Y_{i,t} \sim NB(\mu_{i,t}, \theta_{i,t}) \qquad \text{eq. 1}$$

Under this assumption, the distribution of the missing delays over time and location $Y_{i,t}^m$ can be imputed from the parametric approximation of the distribution of the observed delays $Y_{i,t}^o$ over the same time and location.

We further assume that for a short period of time (τ) before the latest date of imputation d which comprises consecutive reporting dates with lags $0,1,..u$, such as $\tau = d-u, d-u-1,..d$, the reporting delay distribution conditional to DOR for the Spanish dataset remains relatively homogeneous. However, aiming to account for the weekly reporting patterns commonly present in infectious diseases reporting (e.g. fewer reporting during weekends), we also assume that the expectation of the one-week backward reporting delay distribution is affected by the day of the week. We model this dependency by using a categorical predictor w for the report day being a weekday (Monday to Friday) or a weekend (Saturday or Sunday). For each reporting day d, we first fit a generalized linear model to the observed reporting delay distribution over τ, using the log-link function in eq 1, with the reporting delay distribution modeled as a log-linear equation with expectation for day d :

$$\log(\mu^{o}_{i,d}) \sim \beta w_{i,d} \qquad \text{eq. 2}$$

We then estimate for each day $\hat{\mu}^{o}_{i,d}$ and $\hat{\theta}^{o}_{i,d}$ using maximum likelihood [9]. We also estimate the standard error of the mean of both parameters, $SEM_{\hat{\mu}^{o}_{i,d}}$ and $SEM_{\hat{\theta}^{o}_{i,d}}$.

Using observations over τ, instead of only using observations at d allows us to increase the size of the observed delays at each location for the fitting step, as it becomes a particular limitation for NB regression when only a low number of observed delays (e.g., less than 50) is used, while still adjusting to weekday dependence and week-to-week changes on the reporting delays. Formulating alternative models for weekday dependence in the regression (or including other predictors such as age) can increase the accuracy of the imputation [22]. However, this will likely require further understanding of the reporting process and assumptions that need to be evaluated.

At each reporting day and location there are $m_{i,d}$ individuals with missing DOS. Thus for each reporting day and location we generate a set of $n = m_{i,d}$random samples of missing backward reporting delays by predicting the reporting delays using the model in eq. 1 and eq. 2 and allowing for additional dispersion for the parameters $\mu^{m}_{i,d}$ and $\theta^{m}_{i,d}$, and aiming to reliably estimate the uncertainty around the epidemic trends. Specifically, we model each parameter as having a normal distribution with mean equaling $\hat{\mu}_{i,d}$ and dispersion equaling $\hat{\theta}_{i,d}$, computed previously from the observed delays distribution, and the standard deviation equaling the computed SEM of each estimated parameter from observations.

$$\mu^{m}_{i,d} \sim Normal(mean = \hat{\mu}^{o}_{i,d} \,,\; sd = SEM_{\hat{\mu}^{o}_{i,d}}) \qquad \text{eq 3.}$$

$$\theta^{m}_{i,d} \sim Normal \quad (mean = \hat{\theta}^{o}_{i,d} \,, sd = SEM_{\hat{\theta}^{o}_{i,d}})$$

In our main model, we used a maximum lag u of 2 days (i.e., a 3-days period) to impute the missing delay samples, when the number of available observations for that period was 50 or more; when the number of observations in the previous period was less than 30, we increased the number of observations for the regression by increasing the maximum lag to 6 days (i.e., a 7-days time period) and to 9 days (i.e,, a 10 days period) if pooling from the previous again did not provide at least 30 observations. If the number of available observations after this procedure was smaller than 30 (such as the beginning of the outbreak for Murcia), we imputed the missing delays by subtracting the mean delay computed from the observed cases from the DOR of each case with missing DOS. We resampled 100 times to generate 100 time series of case-counts with complete DOS-DOR for each day and region. The sum of the observed and imputed cases became the total cases used for nowcasting.

This text also addresses comments in B.1 and B.2 (see response below)

B) General comments w.r.t. to the model (in real-time surveillance) as I understood it:

1. Estimating the reporting delay distribution for day t =1, …, T based on the days t-1 and t+1 appears to be questionable out of two reasons:
First, in real-time surveillance the data for T+1 is not available (where T is the current date, I.e., "now"). Second, infectious disease reporting data usually follows strong weekly patterns, and the authors mention this also for the Spanish data. This translates also to the backward delay distribution (e.g., if Sundays have few reported cases, the (average) reporting delays should be bigger on Mondays). It might therefore be problematic to estimate the reporting delay distribution for, e.g., a Sunday by aggregating over the collected data from Saturday-Monday, or for a Tuesday by aggregating over Monday-Wednesday. In future work you might just consider modelling the backward reporting delay distribution based on, e.g., a parametrical statistical (regression) model where you can account, e.g., for changes over time, week-day effects, etc. It is then also possible to perform a formal model statistical model evaluation/selection. You could add this option to the discussion.

Thank you. We agree with the reviewer on this query. By using data from t-1 and t+1 we aimed to use information over a longer period of time (3 days) under the assumption that the delay distribution was relatively homogeneous (therefore the parametric fit would be consistent), which in turn provides a larger sample of observations. However, we understand that particularly using t+1 is misleading, as it would only then allow "nowcast" on one day before the last day with available data. Now we fit only data available before the last day of the nowcast and rewritten the text accordingly. We mainly use observations in a 3-days "rolling" time period comprising the day of nowcasting *d*, as well as *d*-1 and *d*-2 while also including "weekday" dependency by using a categorical variable for the expectation of the predicted/imputed delays.

Now stated in the Methods:

We dynamically estimated the parametric delay distribution at each day by regressing the backward delay distribution from all cases with available DOS pooled over a period of time τ comprising all dates between the day of imputation d and a lag u (i.e., $\tau = d - u, \ldots d$) and using maximum likelihood [9]. Using observations over τ, instead of only using observations at d allows us to increase the size of the observed delays at each location for the fitting step. However, to adjust for variation on the reporting patterns due to day of the week (i.e., reduced reporting during weekends compared to weekdays) as well as different short-term dynamical trends (i.e. increasing/decreasing trend over a week), we modeled the mean delay conditional to the categorical predictor w being the reporting date weekday or weekend:

$$\mu^{o}_{i,\tau} \sim \beta\, w^{o}_{i,\tau}$$

For the main analysis we pooled from a 3 -days period when the number of available observations were 50 or more, and sequentially increased τ to 7 or 10 days if the total number of observations in τ were smaller than 30. When the number of observations included in the longest period of time (10 days) were less than 30, we simply imputed the missing delays by subtracting the observed mean delay from the DOR of each case with incomplete information. Last, for imputing the missing DOS at each reporting day d and aiming to reliably estimating the uncertainty around the epidemic trends, we sampled from a randomly generated negative binomial distribution where $\mu^{m}_{i,d}$ and $\theta^{m}_{i,d}$ were modeled as having both a normal distribution with the mean set to the estimated $\widehat{\mu}^{o}_{i,d}$ and $\widehat{\theta}^{o}_{i,d}$, and the standard deviation set to the standard error of the mean."

And the Supplementary:

We further assume that for a short period of time (τ) before the latest date of imputation d which comprises consecutive reporting dates with lags $0,1,..u$, such as $\tau = d-u, d-u-1,..d$, the reporting delay distribution conditional to DOR for the Spanish dataset remains relatively homogeneous. However, aiming to account for the weekly reporting patterns commonly present in infectious diseases reporting (e.g. fewer reporting during weekends), we also assume that the expectation of the one-week backward reporting delay distribution is affected by the day of the week. We model this dependency by using a categorical predictor w for the report day being a weekday (Monday to Friday) or a weekend (Saturday or Sunday). For each reporting day d, we first fit a generalized linear model to the observed reporting delay distribution over τ, using the log-link function in eq 1, with the reporting delay distribution modeled as a log-linear equation with expectation for day d :

$$\log(\mu^o_{i,d}) \sim \beta w_{i,d} \qquad \text{eq. 2}$$

We then estimate for each day $\hat{\mu}^o_{i,d}$ and $\hat{\theta}^o_{i,d}$ using maximum likelihood [9]. We also estimate the standard error of the mean of both parameters, $SEM_{\hat{\mu}^o_{i,d}}$ and $SEM_{\hat{\theta}^o_{i,d}}$.

Using observations over τ, instead of only using observations at d allows us to increase the size of the observed delays at each location for the fitting step, as it becomes a particular limitation for NB regression when only a low number of observed delays (e.g., less than 50) is used, while still adjusting to weekday dependence and week-to-week changes on the reporting delays. Formulating alternative models for weekday dependence in the regression (or including other predictors such as age) can increase the accuracy of the imputation [22]. However, this will likely require further understanding of the reporting process and assumptions that need to be evaluated.

Also following the comment, we have included this issue in the Discussion

“All the previous limitations highlight that a good performance of the nowcasting approach can only be achieved by adequately specifying the model to account for the actual reporting process in the region that is analyzed. Future approaches including additional terms in the regression models can be explored aiming to better account for the reporting dynamics and the challenge of imputing under a small number of observations, while performing formal evaluation procedures can help to support selection of most accurate models [22].”

2. The different approaches to model the reporting delay distribution for (3 consecutive) days with smaller than 50 reported cases compared to days with more than 50 reported cases raises some questions (this seems to be mainly relevant for Murcia). You write that theta is set to 1 for such days, and that this implies that P(d=a)>P(D=b), for all aP(D=1). This seems to be a questionable assumption, as reporting delay distributions usually have a peak of probability mass at a specific delay >0, as also seen in the data (Figure S1). Furthermore, I am irritated by the estimated delay distribution for Murcia for March 8 (Figure S1). The reported cases from Match 7-March 9 seem to be <50 (Figure 1), but the estimated delay distribution seems to have biggest probability mass for a delay of d=2. This seems to be inconsistent with the described imputation model. I do not think that this aspect plays a major role for the general results, but presented results should be consistent with the described modelling approach! Of note: A more general statistical model could also help with the problems arising on days with only few observations.

Thank you. We have now used a simpler approach to deal with a small number of observations by using observations over longer periods (7 days and 10 days) when those over 3 days (or 7) are not sufficient (i.e., less than 30). When the number of observations pooled from a 10 days period was smaller than 30 (i.e., at the beginning of the outbreak in Murcia), we simply imputed the delay by backshifting by the mean observed delay.

"For the main analysis we pooled from a 3 -days period when the number of available observations were 50 or more, and sequentially increased τ to 7 or 10 days if the total number of observations in τ were smaller than 30. When the number of observations included in the longest period of time (10 days) were less than 30, we simply imputed the missing delays by subtracting the observed mean delay from the DOR of each case with incomplete information."

This limitation is also addressed in the Discussion Section

*Future approaches including additional terms in the regression models can be explored aiming to better account for the reporting dynamics and the challenge of imputing under a small number of observations, while performing formal evaluation procedures can help to support selection of most accurate models [22]***.**

C) Reporting of the results:
- Caption in Figure S2 C,H seems to be wrong, the model is not more flexible than the revised main model.

We have changed the caption accordingly:

"C,H) randomly masking 40% of available reporting delays and estimating from a 7-day window of observations and allowing additional variance for the dispersion parameter D,I) randomly masking 40% of available reporting delays and using a single value for the mean in the negative binomial,"

D) Code:
The code in the GitHub repository page does still not correspond to the imputation model you are describing in the manuscript (as of July, 27, 2021)!

Thank you. We have updated the code accordingly to this revised version

Nowcasting:
- Thanks for showing the results of your nowcasting method for additional days, this helps to judge the performance of the model better and the finding of a bias due to short-term changes in reporting activities on weekends is interesting and important to discuss.

Thank you.

- I agree with the conclusion that such biases complicate the interpretation of daily results, and I think it is good that this aspect is now being discussed. Your formulation "when there are large daily changes in the reporting case counts, such as that observed related to weekends, which might preclude public health action based on daily estimates" is however, a bit vague and seems to be grammatically wrong.

We have now changed the statement as follows:

"(...) when the difference in the number of reported cases between consecutive days is substantially large, as for example, when there is a substantial reduction in case reporting during weekends. This limitation might preclude public health action based on day-to-day changes of the estimated number of infections; instead, changes during larger periods should be evaluated."

- In addition, it might be worth mentioning that consistent patterns in the reporting activity can also be considered in more refined nowcasting-models. This can, e.g., be done by letting the probability of being reported on day t+d (d=1, …, D) for an individual with disease onset at day t vary based on the weekday of day t+d and estimating those weekday effects within the nowcasting model. In the NobBS model this would

correspond to modelling beta_td instead of only beta_d, e.g., based on a parametrical model that accounts for weekday changes.

Thank you. We have included the following in the limitations paragraph in the discussion:

"Alternatively, more refined nowcasting models dealing with the forward reporting dynamics [22] could improve the reliability of the estimates."

- I think you should mention in the discussion that a good performance of a nowcasting approach can only be achieved when the model for estimating the reporting delays is adequately specified to account for the actual reporting process in the region that is analyzed.

Thank you for raising this important point which we have also realized. Now, in the discussion the following is included:

"All the previous limitations highlight that a good performance of the nowcasting approach can only be achieved by adequately specifying the model to account for the actual reporting process in the region that is analyzed."

- Minor comment: In supp-note S3 you write "As shown in Figure 1 in the main analysis": This should probably refer to Figure 2.

Corrected

Estimation of R(t), new Figure 3:
- please add telling headings to the sub-figures and a legend to the plots. Use unique or reasonably shared colours for the different estimates of R(t), it is confusing that there are two blue lines

We have now redesigned the panel and included headings and legend to clarify

***Figure 3**. Reproductive numbers estimated from nowcasted cases by DOS vs observed cases by DOR using Cori et al (A,C) and Wallinga and Teunis (B,D) approaches for Madrid and Murcia, March 1-April 09, 2020.*

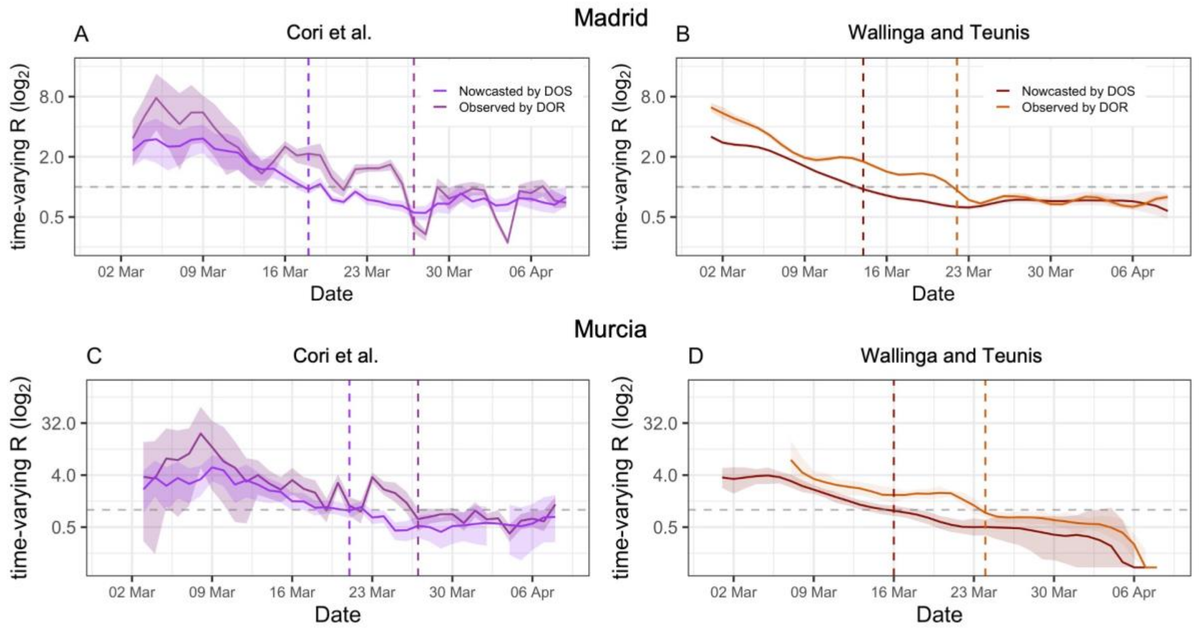

Showing the reproductive numbers estimated for Madrid (A,B) and Murcia (C,D). Estimates were computed using Cori et al (A,C) or Wallinga and Teunis (B,D) approaches and either nowcasted cases by DOS vs observed cases by DOR. A generation interval of mean 5 (1.9 SD) was used. Log_2scale for the y-axis is used to facilitate visualization.

- why does the Walinga-Teunis based estimate for Murcia based on DOR (?, blue line in the new Fig 3D) ends at April 4? n addition, I find it surprising that the two estimates in 3D are so similar (until around March 25), do you have any explanation for this? Is this sub-figure and/or the caption correct or is this the estimate based on all available DOS without imputation and nowcasting?

Thank you very much. This was a bug/error in the code/computation. Rt estimates cannot be obtained from 01.03.2021 to 06.03.2021 using cases by DOR (due lack of reported cases) in Murcia, and we have not shifted the first (on the 7th) estimates correctly for the plot. Both previous points are addressed. Please see the new plot 3D, previous point.

Discussion

- You mention the central limitation of the case reporting data of a potentially time-varying dark-figure in reported cases and write "However, Rt estimates remain unbiased if the proportion of incomplete observations remains time invariant [17,25], as it is likely the case in our analysis." It is, however, not clear why the proportion of underreporting is "likely time invariant". This should be either motivated more clearly or removed from the discussion.
In Supp. Note 4 you write that "a 50% lower ascertainment occurring during the initial period of analysis […] is a plausible change, especially for the first weeks of transmission of COVID-19". In addition, the wording "proportion of incomplete observations" appears to be unclear in the context of nowcasting. From my understanding the issue is about the proportion of missing observations/cases (underreporting) and not incomplete observations (i.e., cases without disease onset information).

We have now removed this statement and reworded the sentence for accuracy in the discussion:

"Major changes in case reporting will bias the R_t estimates [23,24]. However, R_t estimates remain unbiased if the proportion of unascertained or underreported observations remains time invariant [17,25]."

General comments
A) You should revise the entire manuscript and supplementary texts for clarity of wording, correct use of statistical terms, and accurate definition of technical terms and mathematical/statistical variables. Please ensure that the captions of the figures are correct and self-explanatory, that colour choices are consistent and comprehensible, and that each figure has a legend.

Noted with thanks.

Besides the aspects I mentioned already above (e.g., mathematical model for reporting delays and various formulations), I found the following formulations in the current version of the main text to be unclear:

Methods, Step 1: Imputation of missing data:
- Despite my comment on the last version of the manuscript, you continue to use the term "sampling error" wrong. You write: "We resampled 100 times to generate 100 time series of cases with complete DOS-DOR for each region , allowing mu_it and sigma_it to vary randomly under a normal distribution with mean set to their estimated values, and standard deviation set to the sampling error". The sampling error is usually defined as "the difference between a sample statistic used to estimate a population parameter and the actual but unknown value of the parameter". This error cannot be estimated base on a single dataset. I think you should replace this by the "standard error of the estimate".

We agree with the reviewer. Corrected:

"we sampled from a randomly generated negative binomial distribution where $\mu_{i,d}^{m}$ and $\theta_{i,d}^{m}$ were modeled as having both a normal distribution with the mean set to the estimated $\hat{\mu}_{i,d}^{o}$ and $\hat{\theta}_{i,d}^{o}$, and the standard deviation set to the standard error of the mean."

- For the third imputation model in the sensitivity analysis, you write: "Third, we masked DOS in a random 40% of cases and then imputed DOS for all cases reported at any given t and then imputed the same date for all cases reported at any given t as the difference between the observed mean delay and the reported date". This is unclear and the formulation "difference between the observed mean delay and the reported date" does not really make sense. You could describe this approach more clearly as an imputation by "subtracting the observed mean reporting delay from the reporting date for each case". Here you might also define the term "backshift" that you are using later in the manuscript.

Thank you. Rewritten as advised:

"Third, we masked DOS in a random 40% sample of cases and then imputed the missing DOS by subtracting the observed mean reporting delay from the reporting date for each case [17] (a procedure hereinafter referred to as backshifting)"

Methods, Step 2: Nowcasting the epidemic curve
- Please define your mathematical notation! What does lower-case t stand for? In the previous paragraph on imputation, you used it for each day in the whole analysis period. For describing the problem of nowcasting, it should probably stand for the last day in the observation period, this is often referred to as capital T.

We have now edited it for consistency. We use now in this paragraph the term *d* for the day over the analysis period.

"The model underlying NobBS assumes that the observed number of cases at location i and day $d = 1, \ldots D$ with delay $l = 0, \ldots L$ follows a negative binomial distribution with parameters $p_{d,l}$ (probability of failure) and r (number of failures before stopping), such as:

$$n_{i,d,l} \sim NB(r, p_{i,d,l}) \qquad \text{eq. 4}$$

$$p_{i,d,l} = r/(r + \lambda_{i,d,l}) \qquad \text{eq. 5}$$

where $\lambda_{d,l}$ is the mean which is modeled as a log-linear equation depending on α_d which is the true signal and β_l, the probability of delay l, adapted from [1]:

$$log(\lambda_{i,d,l}) = \alpha_{i,d} + log(\beta_{i,l}) \qquad \text{eq. 6}$$

As prior distributions on r,α_d,β_l we used the weakly informative priors used by NobBS by default: r is a Gamma distribution with parameters shape and rate $Gamma\ (60{,}20)$ to reflect moderate deviation from a Poisson distribution; α_d is a first-order geometric random walk, aiming to capture the temporal autocorrelation of infections:

$$\alpha_{i,1} = N(0{,}0.001) \qquad \text{eq. 7}$$

$$\alpha_{i,d>1} \sim N(\alpha_{i,d-1}, \tau_\alpha^2)$$

with parameter τ_α^2 modeled with the prior $\tau_\alpha^2 \sim Gamma\ (0.001{,}0.01)$; last, $\beta_{i,l}$, which a probability vector of length L (maximum delay), is a Dirichlet distribution where:

$$\beta_{i,l} \sim Dir(\theta) \qquad \text{eq. 8}$$

$$\theta_{i,l} = \theta_{i,l}, \ldots \theta_{i,L}$$

"

Results, Nowcasting the epidemic curve:
- You write "However, when there is a high proportion of missing data the nowcasting procedure is sensitive to large changes in reported cases". It is not clear what this means. Being sensitive to large changes is not necessarily something bad. I guess you mean that that large and sudden changes in reported case numbers close to the end of the current observation period (e.g., due to an unmodelled change in reporting activity during weekends), can bias/distort the estimates of your nowcasting procedure.
- Directly in the following you write "the bias decreases as more data becomes available". Here it is not clear what bias you are referring to and when it decreases. After seeing more data from consecutive days?

Thank you. We have edited accordingly, and removed the last statement:

"However, the nowcasting procedure can be biased when there is a high proportion of missing data and sudden changes in the reported case numbers close to the end of the current observation (Section S3)."

- In the last sentence you write: "Further, the occurrence of major changes in ascertainment rates relative to the true epidemic trends might bias the nowcast estimates". I am not exactly sure what you mean by "relative to the true epidemic trends".

We have removed the statement for clarity;

"Further, major changes in ascertainment and/or reporting rates due can substantially bias the nowcast estimates"

Results, Estimation of R(t)

- "The precision of Rt increased over time when more information became available". Please be more precise what you mean by more information. Is it more reported disease onset dates and less imputation or just a bigger number of cases (higher incidence)? Or both?

Thank you. Here we refer to more observed cases over time. We have changed the following for clarification:

"The precision of R_t increased over time when more observed cases became available over time, as seen in the third row of Figure 1 for both regions"

- Please add a legend to Fig 3!

Added.

B) Please update the GitHub repository with the code that you are actually using in your analysis, currently it seems to be outdated and your data availability statement is therefore not correct!

Updated.
