# Supplementary material for: Near real-time surveillance of the SARS-CoV-2 epidemic with incomplete data
Source: PLoS Comput Biol. 2022 Mar 31;18(3):e1009964. doi: 10.1371/journal.pcbi.1009964 (PMC9004750; doi:10.1371/journal.pcbi.1009964)
Supplement: Attachment — Submitted filename: Response_Nowcasting_Plos_Comp_Bio.docx [file pcbi.1009964.s014.docx]

Dear editors Dr B M Althouse and Dr T Britton,

Thank you for allowing us to send a revised version of our manuscript for further review in *PLOS Computational Biology*. Thanks to the reviewers’ detailed and constructive feedback and insights we believe that the quality of our revised manuscript has significantly improved.

Given the interdisciplinary nature of our manuscript and the wide range of potential readers, from public health technicians to applied statisticians, we have focused on highlighting the applicability of our methodology in current disease surveillance systems, at the request of reviewers, while making sure that the underlying statistical approaches are robust, clear, and concise.

Please find detailed responses to all concerns raised by reviewers and a revised version of our manuscript with changes highlighted below.

Sincerely, on behalf of all co-authors,

Pablo M De Salazar

Responses to reviewers’ questions

*Reviewer #1: This paper proposed a two-step approach to reconstruct the epidemic curve in nearly real time, firstly imputing the cases with missing symptom onset dates based on those with complete data and secondly, nowcasting the epidemic curve to address the right censoring issue. This method addresses a very common issue faced by infectious disease epidemiologists worldwide, especially during the ongoing outbreak of COVID-19. The paper is well written and clearly presented. I have two comments that I hope the authors can address during revision.*

We appreciate the reviewer’s comments.

*First, since an important application of the method is to estimate Rt in real time, I would like to see a comparison of the Rt estimates with and without the proposed method. Currently, many reported Rt values for COVID-19 were based on the observed number of cases by symptom onset date (e.g., early papers based on data from Wuhan: PMID: 32275295 and 32674112). It will be good to make a direct comparison in this paper and discuss the implications for current COVID-19 surveillance.*

We agree with the reviewer that a major issue on identifying relationships between disease incidence and interventions or other factors/drivers of transmission is the date used to reconstruct the appropriate epidemic curves at a daily resolution. Our results show that major biases can be introduced when using the date of report instead of the date of symptoms onset to calculate Rt values. This is shown in the sensitivity analysis, in the Supplementary materials Section S4, Figure S7. To make this clearer on the main manuscript, we have now included an additional figure (Figure 3, main text) which illustrates the major differences using our proposed approach vs using the date of report. We have also included a more detailed description of the findings in the results section and clarification in the discussion section. Specifically, the following lines have been added to the results:

*“R_t_ estimates computed using nowcasted curves show an earlier reduction of R_t_ than those obtained from raw case counts by DOR as seen in Figure 3; further, R_t_ estimates from case counts by DOR showed significantly higher values followed by a steeper slope for both approaches, and more noise when using Cori’s approach compared to the estimates from the nowcasted curves (further details can be found in supplementary material, Section S4, Figure S5).”*

The following figure has been added to the revised version of the main manuscript, in the results section*:*

***Figure 3****. Reproductive numbers estimated from nowcasted cases by DOS (red/purple) vs observed by DOR (black) using Cori et al (A, C) and Wallinga and Teunis (B,D) approaches assuming a generation interval of mean 5 (1.9 SD), for Madrid and Murcia, March 1-April 16, 2020*

*Madrid	Murcia*

And the following lines have been added to the discussion:

*“Nowcasted case counts were more accurate and consistent with true transmission than the unadjusted curve by DOR (as documented using complete data that became available after the study period). For example, our approach could identify SARS-CoV-2 epidemic control by lockdown in Spain almost a week earlier than when using reported cases by DOR as the transmission proxy”*

*Second, the authors pointed out one limitation of their approach is that the ascertainment rate does not change significantly over time. But the testing capacity in many countries has changed a lot since the outbreak. It will be good to use simulations to understand the impact of the violations of this assumption so that we can better interpret the results when applying their method.*

At the reviewer's recommendation, we have now included a sensitivity analysis in the Supplementary materials (see Section S4, Figure S8) aiming at illustrating the impact of likely changes in ascertainment on both the nowcasting and the subsequent estimation of R(t). Specifically, we have included an evaluation of how the nowcast, and the subsequent R(t) time series would change if a reduced (50% lower) ascertainment occurred during the early period of our analysis. We believe this may be a plausible estimate, especially in the first weeks of transmission of COVID-19. As can be seen in our assessments, the results are comparable. We argue in our manuscript, however, that for the time period and regions of analysis of our work, ascertainment rates should not be expected to significantly change. Thus, we believe that R(t) point estimates found using our assumptions remain accurate if the rate of incomplete observations remains constant. Previous work [1] has evaluated the issue of incomplete observations for both approaches, and more recently for COVID-19 [2]; we have included a clarification on this matter in the discussion and referenced the relevant work:

*“Fourth, our approach requires that the degree of underreporting of cases be relatively constant over time. Major changes in case reporting will bias the R_t_ estimates* [*[23]*](https://paperpile.com/c/k3VeiC/tYfR)*. However, R_t_ estimates remain unbiased if the amount of incomplete observations remains relatively time invariant* [*[17,24]*](https://paperpile.com/c/k3VeiC/251Wt+J4Zs)*, as it is likely the case in our analysis.”*

The following analysis and figures have been added to the Supplementary:

***Sensitivity of*** $\boldsymbol{R}_{\boldsymbol{t}}$ ***to changes on ascertainment rate***

*For our dataset we do not expect to see major changes in ascertainment rates because clinical and surveillance procedures, as well as capacities did not substantially change during the analysed periods (first two months of the pandemic). However, to illustrate the impact of potential changes in ascertainment on the nowcasting and the subsequent estimation of R(t) on our procedure, we evaluated how a major drop (a 50% lower ascertainment) occuring during the initial period of analysis would impact the estimates. This is a plausible change, especially for the first weeks of transmission of COVID-19. The overall trends of the nowcast curves, time of peak transmission and* $R_{t}$ *were relatively unaffected when simulating a change in ascertainment rate for Madrid, where the true transmission process included a narrow peak with sharp increase and decline, but affected the peak of transmission and consequent* $R_{t}$ *estimation for Murcia, where the true transmission signal showed a wider period between the increase and decline of cases.*

***Figure S8****. Epidemic curves and Rt estimates using the data available during the the intermediate analysis of the initial SARS-CoV-2 outbreak in the regions of Madrid and Murcia, Spain, March 1-April 9, 2020 (A, D and G, J) and comparison of the same estimate approach after randomly subtracting 50% of cases during the first 2 weeks (without including peak transmission, B,E and H,K) and during the first 4 weeks (including peak transmission, C,F and I,L)*

*Madrid*

*Full ascertainment	50% ascert., first 2 weeks	50% ascert., first 4 weeks*

*Murcia*

*Full ascertainment	50% ascert., first 2 weeks	50% ascert., first 4 weeks*

*DOR: date of report; Lines are median estimates, ribbons span 2.5 and 97.5 percentiles. Vertical lines indicate the day when R_t_ <1 (red dashed line for WT, purple dashed line for C)*

References used in this response:

1. White, L. F., & Pagano, M. (2010). Reporting errors in infectious disease outbreaks, with an application to Pandemic Influenza A/H1N1. *Epidemiologic Perspectives & Innovations*, *7*(1), 1-12.
2. Pitzer, V. E., Chitwood, M., Havumaki, J., Menzies, N. A., Perniciaro, S., Warren, J. L., ... & Cohen, T. (2020). The impact of changes in diagnostic testing practices on estimates of COVID-19 transmission in the United States. *MedRxiv*.

*Reviewer #2: The authors propose a three-step analysis of COVID-19 case reporting data for real-time monitoring of the epidemic situation and illustrate it based on the example of surveillance data from the first phase of the pandemic in Spain. The proposed analysis is based on three steps: 1) Imputation of missing dates of symptom onsets (DOS) based on the observed “backward” reporting delay distribution conditional on the date of report (DOR) from all cases with available DOS; 2) Nowcasting of the epidemic curve (number of cases with DOS on a given day) utilizing a previously published Bayesian model (Nowcasting by Bayesian Smoothing); 3) Estimation of the time-varying reproduction number R(t) based on two different approaches. The proposed analysis is illustrated in two Spanish regions at three different time points and results of the procedure for the first two time-points are evaluated based on retrospectively available data (at time point 3) to compare and evaluate different modeling choices.*

*Research on the adequate interpretation of COVID-19 case reporting numbers and real-time surveillance data is a timely and very important topic. Nowcasting can be a valuable analysis tool to adjust reported case numbers for reporting delays. The authors provide an interesting application of such a nowcasting approach, there are however several major aspects that require clarification/revision:*

We appreciate the thorough revision conducted by reviewer 2 as well as their detailed comments.

*1) Missing references and novelty:*

*The proposed three-step analysis is not new; a similar approach of imputation of missing DOS, Bayesian nowcasting, and estimation of R(t) was already proposed and published in Guenther, et al. (2020). There it was shown that it is necessary to adjust for changes in the “forward” reporting delay distribution over time to avoid a bias in the estimation of the epidemic curve.*

Thank you for bringing this oversight on our part to our attention. The rapid changes in the COVID-19 literature make it difficult to stay up to speed. We have now referenced the work mentioned by the reviewer, highlighting similarities and also complementary aspects of our work in a specific paragraph in the discussion.

*“The overall findings of our work are consistent with the evaluation of a similar 3-step approach proposed recently* [*[29]*](https://paperpile.com/c/k3VeiC/4VG7) *and analyses using synthetic data* [*[17]*](https://paperpile.com/c/k3VeiC/J4Zs)*. Nevertheless, we extend the analysis by focusing on, and illustrating, key aspects of the method and assumptions that support further adaptation of the approach to surveillance in other settings; by comparing different regions and periods of analysis; and by providing alternative models for reconstructing the epidemic curve and their evaluation while using existing computational tools.”*

*2) Missing methodological details and modeling choices:*

*2.1. Imputation: the authors model the reporting delay distribution given DOR based on a Negative Binomial model with varying mean per day and region and constant overdispersion parameter per region. Especially in times of low reporting numbers, modeling a single mean parameter per day might yield rather unstable results. On the other hand, the overdispersion of the NegBinom distribution might vary over time as well (as also described by the authors based on empirical data). Did the authors perform any (formal) model selection for the imputation model?*

We would like to thank the reviewer for this comment. As the reviewer points out, some important misspecifications might arise upon this model assumption. Following the reviewer's comment, we have now clarified that our imputation step allows random variation of the mean and the dispersion parameter. Moreover, as sensitivity tests, we assessed two additional alternative formulations, one more and one less flexible compared to the presented constant over-dispersion approach. Specifically, we used two alternative parameterizations for the reporting delay (after masking DOS in a random 40% of cases): a) a single mean parameter $\mu$over days and region (stronger assumption than the main approach) b) mean parameter $\mu_{i,t}$ and dispersion parameter $\theta_{i,t}$ estimated from the distribution of observed delays pooled from $t-\tau$ to $t$ (weaker assumption than the main approach with $\tau$=7). We compared these two alternative approaches to the main one by visual inspection (i.e. if the median and intervals deviated over/under true observed cases). Additionally, we evaluated the prediction using summarizing accuracy measures for the central tendency estimates (MAPE).

Overall, we feel that the more flexible model did not show any significant improvement. The procedure is included in methods and a detailed description and plots in the supplementary material section S3.

The following lines have been added to the methods:

*“We conducted several sensitivity analyses to explore the impact of the method's assumption on the estimates. First, we repeated the analyses after masking DOS (hiding original data as missing values) in a random 10% and a random 40% of reported cases. Second, we used two alternative parameterizations for the reporting delay (after masking DOS in a random 40% of cases): a) a single mean parameter* $\mu$*over days and region(stronger assumption than the main approach) b) mean parameter* $\mu_{i,t}$ *and dispersion parameter* $\theta_{i,t}$ *estimated from the distribution of observed delays pooled from* $t-\tau$ *to* $t$ *(weaker assumption than the main approach with* $\tau$*=7).”*

We added the following lines to the results in our revised manuscript:

*“Compared with the main analysis, the more flexible negative binomial model did not result in a significant improvement (see supplementary material, Section S2 Figure S2).”*

And added the following text to the Supplementary Material:

*We further evaluated the accuracy of the imputation model producing 100 epidemic curves after randomly masking 10% and 40% of the cases with available DOS for the later stage and reconstructing the observed curve. As seen when comparing Figure S2 A and F vs B and G, masking increases the uncertainty by widening the range of the possible imputed time series. However, when a significant percentage of cases are censored, the imputation model leads to a smoothing of the epidemic curve estimates, reducing the initial epidemic curve slopes and the global maxima, which might be critical when evaluating narrow point estimates. As expected, smoothing was greater when using less flexible parameterizations. The Mean Absolute Percentage Errors (MAPE) for the main model vs the less and more flexible alternatives were 10.9%, 11.3% and 12.7% respectively for Murcia, and 11.7%, 12.2%, 11.2% for Madrid. Mean delay backshift after randomly masking 40% of the cases provided less accurate estimates, conditional on the noise found at the time series by date of report (Figure S2 E and J). We also have evaluated alternative distributions for the reporting delays, namely the Poisson distribution and the more flexible Conway-Maxwell-Poisson distribution, none of which improved the estimates (data not shown).*

***Figure S2.*** *Plotting the sum of imputed and observed* *epidemic curves (black line median, ribbon 95% CI) in the regions of Madrid and Murcia, Spain, March 1-April 16, 2020, estimated after a) randomly masking 10% of available reporting delays and using the main imputation approach, b) randomly masking 40% of available reporting delays and using the main imputation approach, c) randomly masking 40% of available reporting delays and using a more flexible approach (estimating from 7-days window of observations and allowing additional variance for the dispersion parameter) d) randomly masking 40% of available reporting delays and using a less flexible approach (using a single value for the mean in the negative binomial), and e) randomly masking 40% of available reporting delays and imputing by mean delay backshifting. Blue columns represent true observed case counts by day of symptoms onset.*

Madrid	Murcia

We also have evaluated a Poisson distribution and the more flexible Conway-Maxwell-Poisson distribution, none of which showed to improve the reconstructed estimates by visual inspection; we have included a statement on this (see previous inserted text, last paragraph). We believe a more detailed statistical evaluation of the imputation model remains important, but we also think it would go beyond the scope of the manuscript.

*The imputation implies a missing at random assumption and the authors show in Supplemental section S1 that this imputation works quite well when the assumption is fulfilled (DOS missing for randomly selected individuals). In reality, the assumption might however be violated, e.g., if DOS is missing mainly because of pre-symptomatic cases. The current sensitivity analysis does not address this problem, and this should at least be discussed. Furthermore, the possible extent of such a violation might be quantified in a sensitivity analysis by setting the DOS for cases with missing DOS to the DOR or e.g. DOR+2 in a sensitivity analysis.*

We agree with the reviewer that the assumption might not fully hold in reality. Following the reviewer recommendation, we have evaluated in a sensitivity analysis how a plausible deviation from the assumption would affect the accuracy of the reconstruction through the imputation by (a) randomly masking 20% of cases among those with delays shorter than the median delay and by (b) randomly masking 20% of cases among those with delays longer than the median. We chose this sensitivity test because it illustrates well how a moderate and systematic bias on the assumption affects the construction of the epidemic curve. We found that deviation from the assumption weakly skewed the overall trajectory of the curve, though this appeared to be conditional to the amount of data missing that needs to be imputed. The sensitivity has been included in the methods and results, with a detailed description in the Supplementary Material Section S2, Figure S3 and a clarification of the limitations of the approach in the discussion section. The following was added to the methods:

*“Last, we evaluated how deviations from the missing-at-random assumption would impact the imputation by randomly masking 20% cases with delays shorter than the median delay from that analysis period, or masking those cases with delays longer than the median.”*

We added the following to the results on the revised manuscript:

*“Additionally, the curve was skewed when the assumption of missing-at-random was violated (Section S2, Figure S3).”*

And added the following clarification on limitations of the approach to the discussion:

*“First, its validity relies on the assumption that the date of symptoms onset is missing at random, that reporting delays can be approximated using a parametric approach, and that the available historical data are sufficient to parameterize the unknown reporting delay. These assumptions might be violated in many different ways depending on factors such as the accuracy, quality or procedures of the surveillance systems. However, our sensitivity analyses indicate that the overall trajectory of the epidemic curve was relatively robust to small-to-moderate departures from these assumptions. Second, our approach underperforms when little information is available for training the nowcasting algorithm, including good estimates of the reporting delay distribution.* *”*

We added the following to the Supplementary Material Section S2, Figure S3:

*Last, we evaluated how major deviations from the missing-at-random assumptions would impact the imputation step, by reconstructing the true observed curve after a) randomly masking 20% cases among those with delays shorter than the median delay , or b) randomly masking 20% cases among those with delays longer than the median. The reconstruction is a skewed version of the smoothing that appears under the missing-at-random hypothesis. We observed that the imputed estimates were biased in the opposite direction of the bias in the masked data: when “missing” (masked) cases were mostly among those with longer reporting delay, the imputed curves underestimated cases of the increasing trend and overestimated on the decreasing trend (Figure S3 A and C); when missing cases were among those with shorter delays, the increasing trend was overestimated and the decreasing underestimate (Figure S3 B and D).*

***Figure S3.*** *Plotting the sum of imputed and observed* *epidemic curves (black line median, ribbon 95% CI) in the regions of Madrid and Murcia, Spain, March 1-April 16, 2020, estimated after a) randomly masking 20% of available reporting delays among cases with delays longer than the median (A and B) and b) randomly masking 20% of available reporting delays among cases with delays shorter than the median (C and D). Blue columns represent true observed case counts by day of symptoms onset.*

*Madrid	Murcia*

*2.2. Nowcasting: the description of the nowcasting model is very brief and hard for me to understand (even when additionally consulting the original NobBS publication). The selection and specification of the hyperparameters and priors is not motivated and incomplete. What was the maximum considered delay? Does the model assume a delay distribution that is independent of day t in the whole moving window? It is unclear to me what exactly the parameters alpha_t and beta_d as defined by the authors are and what assumptions the mentioned uniform priors imply. The notation and/or the prior do not correspond to the original publication of NobBS?*

We have now added a detailed section (Section S1, *Nowcasting model*) in the supplementary on the nowcasting model including a description of the prior distributions of the negative binomial parameters used in the main analysis

*“Section S1. Additional information on model specification*

*Nowcasting model*

*We used NobBS to nowcast the number of yet unreported cases with DOS at t that would be eventually reported under the assumption that the forward-delay conditional on DOS occurs as a negative binomial process. NobBS uses historical information on the reporting delay to predict the number of not-yet-reported cases in the present using a log-linear model of the number of cases. The implementation of NobBS requires the specification of 1) a sliding window for the time-varying reporting delay, 2) a maximum delay allowed for the window (D). We set up the moving window as the 75% of the total number of days in each of the periods of analysis and the maximum delay as the moving window-1 day.*

*The model underlying NobBS assumes that the number of cases at time* $t$ *with delay* $d$ *follows a negative binomial distribution with parameters* $p_{t,d}$ *(probability of failure) and* $r$ *(number of failures before stopping), such as:*

$n_{t,d}$*~*$NB(r,p_{t,d})$

$$p_{t,d}=r/(r+\lambda_{t,d})$$

*where* $\lambda_{t,d}$ *is the mean which is modeled as a log-linear equation depending on* $\alpha_{t}$ *which is the true signal and* $\beta_{d}$*, the probability of delay* $d,$*adapted from [1 ]:*

$${log(\lambda}_{t,d})=\alpha_{t}+log(\beta_{d})$$

*As Prior distributions on* $r$*,*$\alpha_{t}$*,*$\beta_{d}$ *we used the weakly informative priors used by NobBS by default:* $r$ *is a Gamma distribution with parameters shape and rate*$Gamma (60,20)$ *to reflect moderate deviation from Poisson;* $\alpha_{t}$*is a first-order geometric random walk, aiming to capture the temporal autocorrelation of infections:*

$$\alpha_{1}= N(0,0.001)$$

$\alpha_{t>1}$*~* $N(\alpha_{1}, \tau_{\alpha}^{2}$*)*

*with parameter* $\tau_{\alpha}^{2}$ *modeled with the prior* $\tau_{\alpha}^{2}$ *~*$Gamma (0.001,0.01)$*; last,* $\beta_{d}$*,which a probability vector of length D (maximum delay), is a Dirichlet distribution of where:*

$\beta_{d}$*~* $Dir(\theta)$

$$\theta_{d}=\theta_{0},...\theta_{D}$$

*The NobBS method can be implemented in R using the NobBs package (v1.2), which compiles in JAGS using the rjags package (v4.10).”*

*2.3. Estimation of R(t). The authors estimate R(t) based on the methods of Wallinga and Teunis and Corri et al. and note correctly that they are conceptually different. It would be helpful to provide information on the correct interpretation of the different estimated R(t)'s at a given time point t. Is there any advantage of one approach over the other?*

We have provided clarifications on the interpretation of the different R(t) approaches in the methods section:

*“Because WT and C use different forward and backward-looking information computational approaches, respectively, a relative delay approximating the mean generation interval of WT relative to C is to be expected* [*[17]*](https://paperpile.com/c/k3VeiC/J4Zs)*. Further, downward biased WT estimates are expected for the last period of nowcast of length equivalent to the generation time, as they would lack sufficient data for computation.”*

We added the following to the results on the revised manuscript:

*“As expected, R_t_ estimates using the WT method for the last period of analysis were biased downward, which precludes its use for the last period of length equal to the generation interval.”*

We have also referenced previous work where the issue has been thoroughly evaluated, and where the advantages of one method over the other one have been discussed.

*Two further questions/remarks:*

*How was the generation time/serial interval distribution specified?*

We have clarified the details on the generation time distribution in the supplementary material Section S1,

***Specifications of the generation interval distribution.***

*“We estimated the time-varying reproduction number* $R_{t,\tau}$*, where* $\tau$*is the window over which it is computed, using two approaches implemented in the R package epiEstim (v2.2.1): Wallinga and Teunis* [*[13,19]*](https://paperpile.com/c/k3VeiC/2HkFx+us4dh)*) (WT) and Cori et al. (C)* [*[13,19]*](https://paperpile.com/c/k3VeiC/2HkFx+us4dh)*. The package allows to reconstruct the reproductive number time series by using the epidemiological curve and a parametric approximation of the generation time (the time between a primary and a secondary case infection) with uncertainty in its distribution. The distribution is assumed to be Gamma, which mean* $\mu_{si}$ *and standard deviation* $\sigma_{si}$ *are also allowed to vary at a truncated normal distribution. To parameterize the Cori et al model in the main analysis, we used the median and standard deviation estimates from* [*[20]*](https://paperpile.com/c/k3VeiC/zFntk) *and allowed for additional uncertainty: average* $\mu_{gi}$ *of 5 days (sd 1, min 2, max 7) and with an average* $\sigma_{gi}$ *of 1.9 (sd 0.5, min 1, max 3). In sensitivity analyses, we used estimates of the mean and sd of the serial interval from* [*[21]*](https://paperpile.com/c/k3VeiC/zFntk) *, assuming an average* $\mu_{gi}$*of 7.5 days (sd 2, min 4, max 11) and an average* $\sigma_{gi}$ *of 3.4 (sd 1, min 1, max 7). However, the current WT estimate procedure in epiEstim does not allow the additional uncertainty around* $\mu_{si}$*and* $\sigma_{si}$*, thus we only used single values. Small-to-moderate deviations from the proposed parameterization did not change the trends of the time series, the time to become <1 and the uncertainty of the* $R_{t}$*estimates for each of the distributions.*

*We set up* $\tau$ *to one day as all epidemic curves included sufficient number of incident cases at all time points for accurate estimates. We computed* $R_{t}$ *from the 10,000 NobBS samples to produce the mean and 95% credibility interval of the* $R_{t}$ *. For computational ease, we used a random sample of size 100 from the 10,000 samples since results did not materially change with a greater sample.”*

*Because its definition is “forward looking”, the Wallinga and Teunis method is biased downwards for all days t for which no information on the number of disease onsets on day t+d is available for all number of days d that have a relevant probability mass in the generation time distribution (e.g., because t+d>current day T). For those days t it appears not to be meaningful to report the estimated R(t) based on WT.*

Thank you. We agree with the reviewer. As this comment relates to previous comment 2.3, please refer to the response we have provided.

*3) Study design:*

*How and why did the authors choose the 3 specific time-points for showing the results of the nowcast? For the sake of illustration this might be a reasonable choice, but a thorough evaluation of the performance of the proposed approach should be made based on nowcasts for every day in the study period (i.e., comparison with “retrospective truth”). In this case coverage frequencies of prediction intervals can be investigated and systematic biases might be detectable. The performance of different model specifications might also be compared quantitatively, e.g. based on scoring rules.*

We selected the time-point for analysis because we aimed to illustrate the application and limitations of the framework in a real-world real-time case-study. The time points selected are indeed those when data was submitted to the centralized surveillance system in Spain. Submission of reports is an additional challenge in real-time surveillance,but considering that 1) it mainly reflects a low quality or lack of appropriate implementation of basic surveillance procedures 2) the processes behind are likely either non generalizable or uninformative to other settings and 3) they are mostly solved by improving surveillance systems, we did not consider that the problem should be approached using modeling.

Therefore, we decided to frame our work as “near real-time” instead of truly “real-time” and used the temporal scale of available submissions for selecting the time points of analysis. We recognize that this requires clarification and we have done so in the methods:

*“We chose these three periods because their ending points correspond to distinct times when decisions about epidemic control were considered in Spain, and thus the limitations arising from the availability of data were especially relevant at those points.”*

And among the limitations in the discussion section:

“*Second, our approach underperforms when little information is available for training the nowcasting algorithm, including good estimates of the reporting delay distribution. This is particularly important in the early stages of emerging outbreaks, when deficits among surveillance procedures, such as challenges on data submission to surveillance systems, will heavily impact the availability of data.”*

Nevertheless, aiming to tackle the specific concern of this reviewer explained in the next point (see below), we have added a sensitivity analysis which we believe helps to illustrate and clarify it.

*4) Performance of analysis/nowcasting model:*

*the available information on the performance of the proposed analysis is insufficient. Especially around the first time-point in the Madrid region the estimated epidemic curve appears to be strongly biased upwards and the (retrospectively observable) decrease in the epidemic curve is not identified by nowcasting. This appears to be the most interesting time point with respect to real-time surveillance. To adequately judge the performance of the proposed analysis approach and its value for real-time surveillance a more formal quantitative evaluation would be necessary (see point 3.) Where does this strong bias at time point 1 come from, is it due to changes in the reporting delay distribution over time (e.g., increased reporting speed due to less cases/fewer workload for the health authorities)?*

*At what day is the peak in the epidemic curve in Madrid around March 15 identified based on the nowcasting?*

Thank you. As explained in the previous point, our analysis is not only limited by challenges addressed in the approach but also because of the availability of data at each time point, which itself depends on the surveillance procedures at the time. To judge the performance of the nowcast methods at the specific time point of interest in a narrower time scale, and following reviewers suggestion, we reconstructed the epidemic curve at each day for a period following the first time point (27 March). Because the analysis cannot be performed only using the data available in the first period (as it ends by March 27), we used the data available in the second period but only for the specific dates March 27 to 30. Note that this second dataset is more complete (relative to the final data available) than the first one for the same time point of analysis (March 27). However, using this second dataset, the reconstructed curve performed very similarly for Madrid and Murcia, as shown in Figure S4 A and E. We also observed that successive increases in the availability of data rapidly corrected the nowcast estimates as soon as March 28 (Figure S4 B and F). We conclude that the lack of accuracy reflected in the first time point is due to insufficient critical available data, which in turn was likely due to an initial deficit on the submission procedures of the surveillance systems in Madrid, as it was corrected later in time.

We have clarified this issue in the methods (see previous point) and results:

*“However, in the early period of analysis for Madrid, with a high proportion of missing data, the nowcasted epidemic curve was higher and peaked later than the curve reconstructed based on later data. In sensitivity (see supplementary material, Section S6) we showed that increasing the availability of data rapidly improved the accuracy of the estimates.”*

; we have addressed this problem in the limitations paragraph in the discussion section (see also previous point):

*“Second, our approach underperforms when little information is available for training the nowcasting algorithm, including good estimates of the reporting delay distribution. This is particularly important in the early stages of emerging outbreaks, when deficits among surveillance procedures, such as challenges on data submission to surveillance systems, will heavily impact the availability of data.”*

; last, we added the following sensitivity section in the Supplementary Material, Section S3:

*“Nowcast performance with increasing available data*

*As shown in Figure 1 in the main analysis, for the early period of analysis in Madrid, the nowcasting approach underperformed well. To judge the performance of the nowcast methods in a narrower time scale, under the hypothesis that increasing availability of data would increase the accuracy, we aimed to reconstruct the epidemic curve at each day for a period following the first time point (27 March). Because the analysis cannot be performed only using the data available in the first period (as it ends by March 27), we used the data available in the second period but only for the specific dates March 27 to 30. Note that this second dataset is more complete (relative to the final data available) than the first one for the same time point of analysis (March 27). However, using this second dataset, the reconstructed curve performed very similarly for Madrid and Murcia, as shown in Figure S4 A and E. We also observed that successive increases in the availability of data rapidly corrected the nowcast estimates as soon as March 28 (Figure S4 B and F). We conclude that the lack of accuracy reflected in the first time point is due to insufficient critical available data, which in turn was likely due to an initial deficit on the submission procedures of the surveillance systems in Madrid, as it was corrected later in time.*

***Figure S4.*** *Evaluation of the reconstructed epidemic curves at each day following March 27 using the data available during the intermediate period of analysis of the initial SARS-CoV-2 outbreak in the regions of Madrid and Murcia, Spain.*

*March 27	March 28	March 29	March 30*

*Showing nowcast estimates (orange) in Madrid and Murcia, observed cases with known date of onset of symptoms for the same period (blue columns), and for comparison with more complete data, those estimated in the late period of analysis (dashed grey line and ribbon); DOS: date of onset of symptoms; DOR: date of report; Lines are median estimates, ribbons span 2.5 and 97.5 percentiles.”*

*5) Nowcasting with different moving window:*

*The presented results with respect to the specification of the moving window are difficult to follow. Based on figure S3 it appears like the maximum considered reporting delay was changed to 5 or 6 days in panel A/D, and for all days before T-5 or T-6 the so far available case counts are considered as final numbers. Based on the data presented in Figure S1 this appears not to be a reasonable analysis and it is therefore not possible to compare the effect of different moving windows for the estimation of the delay distribution. Furthermore, results for the first time point of analysis are missing and would be of main interest.*

Thank you. As suggested, we have performed the analysis using data from the first time point/period in the supplementary material Section S3 (see new Figure S5). We have also revised and edited the text in methods, results and supplementary material to clarify on the use of the moving window and our sensitivity, which includes two alternative approaches: 1) using a fixed, predefined window of 4 weeks, and 2) the alternative of using a more data driven approach (i.e. the moving window changes depending on the changes of the delay distribution). For the second alternative we have also changed the length of the window to be 0.75 of the maximum lengths of observed reporting delay, instead of 0.5 as following reviewers comment we agree the parameter is not sufficiently illustrative/ informative. The following lines has been introduced in the methods for clarification:

*“The implementation of NobBS requires the specification of 1) a sliding window for the time-varying reporting delay, 2) a maximum delay allowed for the window, and 3) a set of priors of the negative binomial distribution parameters. We specified the moving window as the 75% of the total number of days in each period, the maximum delay as the maximum window minus 1 day, and weakly informative priors for the NobBs parameters. Detailed formulation of the nowcast model and prior distributions can be found in supplementary material, section S1.”*

; and the following to improved sensitivity analysis in Supplementary Materials, Section S3:

*“Nowcast estimates using alternative approaches to determine the sliding window*

*We tested the effect of using different strategies for the NobBS window parameter. We compared the strategy used in the main analysis: 1) using a fixed-value sliding window (we selected 28 days), to the alternative observation-driven approach where 2) we changed the length of the sliding window over time depending on the observed delays (we selected the length of the window to be either period comprising 75% or 99% of the observed delay distribution).*

*As expected, the length of the window of analysis changed for each region when using a dynamic approach, with shorter windows using the narrowest criteria (75%) and longer with the widest criteria (99%): for example,for the intermediate period of analysis, we found 20 days of difference in Madrid (26 vs 6) and 15 days in Murcia (20 vs 5, Figure S5). Changing the criteria for determining the window of analysis did not correct for the overestimation of the nowcast curved in the earliest period vs that estimated later in time. Nevertheless, nowcast trajectories were affected by the window of analysis as seen in Figure S5-S6. The length of the window of analysis influenced the accuracy of prediction; as expected, if too short the procedure is unable to nowcast the curve behind the latest day of the window (B and F). A dynamical window including 99% of the backward reporting delay accurately predicted cases later in time (C and G) similar to the main analysis (Figure 2). Finally, imputing by mean delay backshifting of incomplete data led to a less reliable epidemic curve and nowcast estimates (D and H).*

***Figure S5****. Epidemic curves estimated using alternative values for the NobBS sliding window and the data available during the early analysis of the initial SARS-CoV-2 outbreak in the regions of Madrid and Murcia, Spain, March 1-27, 2020, comparison with backshifted epidemic curves by report date, and curves obtained in late period of analysis March 1-April 16 using the main approach.*

*Madrid	Murcia*

*Nowcast estimates in the intermediate period of analysis (yellow lines represent the median and ribbons span the 2.5 and 97.5 percentiles) 1) using a fixed window of 28 days (A and E), 2) using a window that accounts for 75% of the delays available from the latest period of observations (B and F) 3) using a window that account for 99% of the observed delays (C and G ); and 4) imputing through backshifting the report date by mean delay (D and H). Dashed lines and light grey ribbon represent nowcasted cases later in time using the main approach (late period of analysis). Faded blue columns represent observed cases by DOS, faded grey columns represent median imputed cases by DOS; dark grey ribbons (in A, B , C , D , E and F) represent observed plus imputed 2.5 and 97.5 percentiles.*

*6) Unfortunately, there is a lack of data and code to reproduce the analysis. This makes it difficult to understand the analyses performed and hinders the reproducibility and application to data from different regions.*

Thank you. Along with the resubmission we provide the link to the code and a dataset for reproducible results: <https://github.com/pdesalazar/Nowcasting_covid19_Spain>

*Literature*

*F. Günther, A. Bender, K. Katz, H. Küchenhoff, and M. Höhle. Nowcasting the COVID-19 pandemic in Bavaria. Biometrical Journal, 2020. https://doi.org/10.1002/bimj.202000112.*
