# Supplementary material for: Near real-time surveillance of the SARS-CoV-2 epidemic with incomplete data
Source: PLoS Comput Biol. 2022 Mar 31;18(3):e1009964. doi: 10.1371/journal.pcbi.1009964 (PMC9004750; doi:10.1371/journal.pcbi.1009964)
Supplement: Attachment — Submitted filename: Response_v2.docx [file pcbi.1009964.s015.docx]

Dear editors Dr B M Althouse and Dr T Britton,

Thank you for allowing us to send a revised version of our manuscript for further review in *PLOS Computational Biology*. Please find below our responses to the reviewers’ comments.

Sincerely, on behalf of all co-authors,

Pablo M De Salazar

*Reviewer #1: I think the authors misunderstand my first comment. The authors provided new results by applying WT and Cori's method on DOR for comparison. But what I am asking for is a comparison with direct estimate of Rt on DOS without imputation and nowcasting (as have been done in many studies during the pandemic). From Figure 1, we can see that imputation and nowcasting can change the epidemic curves substantially, but the impact on the Rt estimate is not clear. It will be good for the authors to show the difference and discuss the implications on epidemic control.*

*Thank you. We have now computed Rt using cases by DOS without the nowcasting and imputation (censored data). The sensitivity analysis shows that if a high proportion of cases, during the analysis period and/or on specific dates are missed, the computation is not optimal, leading to an underestimation of R_t. For example, for the first period of analysis the estimated time point for when Rt<1 is significantly advanced in time (Figure SX, A,D for both Madrid and Murcia). As more information becomes available (i.e. latest period of analysis) the estimates using available DOS are less biased. Indeed, for our dataset, the added value of the approach is limited to the last days of analysis (~ 7-10 days). We have included this analysis in the Supplementary Information, Section SX*

*“Sensitivity of* $R_{t}$ *when using only observed cases by DOS:*

*To evaluate how performing imputation and nowcasting improved the* $R_{t}$ *estimates, we computed* $R_{t}$ *using only observed cases by DOS and compared to the nowcasted curves using the full procedure for the 3 periods of analysis. As seen in Figure S8, when a high proportion of cases are missed along the period of analysis and/or on a specific date, the computation is not optimal, leading to an underestimation of* $R_{t}$*. For example, for the first period of analysis the estimated time point for* $R_{t}$*<1 using only available cases by DOS is significantly advanced in time (Figure S8 A,D for Madrid and Murcia). As more information is available (i.e. latest period of analysis, Figure S8 C,F ) the estimates using available DOS are less biased.*

**Figure S8.** Reproductive numbers estimated using only available cases by DOS vs. nowcasted curves and curves by report date using the data available during the three periods of analysis the initial SARS-CoV-2 outbreak in the regions of Madrid and Murcia, Spain, March 1-April 9, 2020

Early analysis	Intermediate analysis	Late analysis

(March 1-27)	(March 1-April 9)	(March 1-April 16)

*Madrid*

*Murcia*

Lines are median estimates, ribbons span 2.5 and 97.5 percentiles. Vertical lines indicate the day when *R_t_* <1 (*R_t_* estimated from nowcasted curves are shown in red dashed line for WT, purple dashed line for C; *R_t_* estimated from available cases by DOS are shown in blue).”

*This also has been briefly updated in the methods:*

*“In sensitivity analyses, we used available cases by DOS without imputation and nowcasting (i.e without adjusting for missingness and censoring)”*

*; in the results:*

*“ R_t_ estimates obtained using observed case counts by DOS (i.e., without performing imputation and nowcasting) shifted in both locations towards lower values, particularly for the first periods of analysis, which in turn lead to a 3-6 days earlier estimates of the critical point where R_t_ becomes <1; the estimates improved once more information was available for the last period (Section S4, Figures S7-S8). .”*

*; and in the discussion:*

*“Nowcasted case counts were more accurate and consistent with true transmission than the unadjusted curve by DOS or DOR (as documented using complete data that became available after the study period).”*

*Reviewer #2: Dear Authors,*

*Thank you very much for providing a revised version of the manuscript and for the answers to my previous comments, through which several of my questions were answered. I have, however, some remaining questions/concerns that have to be addressed before I can recommend publication:*

*Despite the additional results and analyses, I am still not convinced about the performance and the added value of the proposed approach in the presented application scenarios, especially for the data in the first period in the Madrid region, which appears to me as the most important with respect to a (near) real-time assessment of the pandemic situation. This is due to the following reasons:*

*1) I am confused by the changes of the nowcast results for Madrid and Murcia on March, 27 (first period) compared to the initial submission. The results in Figure 1 and especially Fig 2A/D do not correspond to the results of Fig 2A/D in the initial submission of the manuscript. The bias in the results is not visible anymore but you did not state any changes with respect to the model or the utilized data in your response to the reviewer comments or the manuscript.*

Thank you. During the process of reviewing the first version of the manuscript, we changed one detail of the imputation model used in the main analysis: in order to produce the original Figure 1, we were not scaling the theta parameter on the negative binomial to the response level. This detail impacted mostly the imputation estimates under a high proportion of missingness, which is the case of the first period of analysis in Madrid. We corrected this issue but we did not include it in the response. We apologize for the inconvenience of not being explicit on this change.

*“We assumed that missing DOS occurred at random with respect to symptom onset date, and that reporting delays conditional on DOR can be modeled over time*$t$*and location* $i$*as a negative binomial distribution with mean parameter* $\mu_{i,t}$ *and dispersion parameter* $\theta_{i, t}$*, which we estimated using maximum likelihood estimation* [*[9]*](https://paperpile.com/c/k3VeiC/4Xck9)*. We resampled 100 times to generate 100 time series of cases with complete DOS-DOR for each region* $i$*, allowing* $\mu_{i,t}$ *and* $\theta_{i,t}$*to vary randomly under a normal distribution with mean set to their estimated values, and standard deviation set to the sampling error. ”*

*2) The results in the new sensitivity analysis (Fig S4) that incorporate more data (since they are based on the data from the second period restricted to what was available until March, 27) appear to be worse than the results presented in (the new) Fig 2A. This is very confusing and the introductory sentences for Supplementary Section S3 do currently not make sense to me. You state "As shown in Figure 1 in the main analysis, for the early period of analysis in Madrid, the nowcasting approach did not perform sufficiently well", but Figure 1/2A do now show quite accurate results. I am, however, not sure where they are actually coming from.*

We thank the reviewer for this comment. In this sensitivity we focused on how the epidemic curve “corrected” from overestimating, which is what we referred to when we say that it did not perform sufficiently well. We have now changed that sentence to the following to be aligned with the main text:

*“As shown in Figure 1 in the main analysis, for the early period of analysis in Madrid, the nowcasting approach slightly overestimated the observed trend.”*

In the next point we further explain what we have done to try to further address the reviewer’s concern

*3) I am skeptical about your interpretation of the results of Figure S4. You state "We also observed that successive increases in the availability of data rapidly corrected the nowcast estimates as soon as March 28 (Figure S4 B and F)." The nowcast of March 29 (Figure S4-C) shows however, a strong bias into the opposite direction (i.e., an underestimation of the epidemic curve). The results from data up until march 27 and up until March 29 are qualitatively very different (and the situation was even worse with the data that was actually available until March 27 with more missing DOS). Altogether, in two out of the four days of the new sensitivity analysis, the 95%-PIs do not cover the retrospective "true" epidemic curve (based on information available at the third period) for several days close to "now". This raises the questions whether the approach would really be helpful in a real-time assessment of the current situation (and is capable "to aid decision-making process" as you state in the discussion) during the first period in Madrid and whether the uncertainty quantification of the method is adequate. Based on these results, and despite the difficulties you have with the availability of the data, I would really recommend doing a proper quantitative evaluation of the performance of the nowcast model over a longer period of time to prove adequate performance. In addition, you should clarify the issues around Figs. 1 and 2 in your manuscript and discuss the problems with the nowcasting approach during the first period in more detail in the manuscript.*

Thanks to this comment we realized that we were overconfident in our model predictions. We have now evaluated with more detail the nowcast estimates over time, and we believe have identified the source of lack of accurate prediction: reporting rates (or number of cases reported by date) change dramatically on weekends. The nowcasting algorithm assumes some degree of smoothness in the reporting rates. We looked at the day of the week when the change of behavior was so abrupt in the predictions (29th March) and it was a Sunday, with very low reported cases both the 28 and 29th March compared to the previous days, and about 50% of missingness. These two combined likely led to the abrupt underpredictions seen on 29th March. To confirm this, we have now extended the analysis which includes the subsequent weekend with a drop in reported cases (4-5 April). Again, the nowcast curve drops on the weekend when comparing to information later on time. Please also note that we have changed the way of plotting the “validation curve” (i.e. the one obtained with more information later in time) to include true observations, imputations and nowcast. Further, please note that now the model is more accurate for 25-27 March; this is because the parameterization that we use now allows more flexibility on the $\theta$. More details are given in the next response.

We believe that, because this limitation (i.e. big drop/increase in reported case counts with high missingness) arises from the data itself, and is likely to arise in other approaches as this weekday noise has been consistently observed across countries and locations; thus, we think that the best way to accommodate it is to make aware decision makers of this limitations, and to either 1) make decisions every third day, not daily, 2) incorporate the information (i.e. the nowcast can underestimate when reporting cases drop with high proportion of missingness) when making decision on weekends. We have now updated Section S3:

*“Nowcast performance in real time with increasing available data*

*As shown in Figure 1 in the main analysis, for the early period of analysis in Madrid, the nowcasting approach slightly overestimated the observed trend. To judge the performance of the nowcast methods in a narrower time scale, under the hypothesis that increasing availability of data would increase the accuracy, we aimed to reconstruct the epidemic curve at each day for a period including the latest dates of the first two periods: 25 March to 8 April. Because the analysis can not be performed only using the data available in the first period (as it ends by March 27), we used the data available in the second period but only for the specific dates March 27 to 30 and April 1 to 8. Note that this second dataset is more complete (relative to the final data available) than the first one for the same time point of analysis (March 27).*

*Using this second dataset, the reconstructed curve performed very similarly for Madrid and Murcia, as shown in Figure S4. However, we observed that when large changes in underlying daily DOR report rate on a specific date affect the model nowcast, especially when in addition a high proportion of cases are missing the DOS. This can be seen for example for Madrid on Saturdays-Sundays ( March 29 or April 4-5), but is less clear for Murcia. This is likely to have implications when the nowcast estimates are used for decision making in real time, as argued in the Discussion section.*

***Figure S4.*** *Evaluation of the reconstructed epidemic curves at each day between March 25- April 8 using the data available during the intermediate period of analysis of the initial SARS-CoV-2 outbreak in the regions of Madrid and Murcia, Spain.*

*Madrid*

*Murcia*

*Nowcast estimates in Madrid and Murcia (orange lines are median estimates, ribbons span 2.5 and 97.5 percentiles, observed cases with known date of onset of symptoms for the late period of analysis (blue columns), cases with imputed date of onset of symptoms for the late period of analysis (grey columns) and nowcasted uncertainty range for the late period of analysis (grey ribbon);Mon: Monday, Tue: Tuesday, Wed. Wednesday, Thu.: Thursday, Fr. : Friday, Sat. : Saturday, Sun.: Sunday.”*

And we have addressed this comment in the methods:

*“Further, we evaluated the nowcast performance in more detail by assessing the changes on the nowcast estimates each day between March 25- April 8, 2020.”*

; in the results:

*“However, when there is a high proportion of missing data the nowcasting procedure is sensitive to large changes in reported cases; the bias decreases as more data becomes available (Section S3).”*

; and in the discussion:

*“Second, our approach underperforms when little information is available for training the nowcasting algorithm, especially when good estimates of the reporting delay distribution cannot be obtained. This limitation might be particularly important a) in the early stages of an emerging outbreak, when deficits among surveillance procedures, such as challenges in data submission to surveillance systems, may heavily impact the availability and consistency of data over time, and b) when there are large daily changes in the reporting case counts, such as that observed related to weekends, which might preclude public health action based on daily estimates. ”*

*Some additional comments with respect to the imputation of missing DOS:*

*4) Based on the description of the imputation model in the manuscript, I still do not completely understand what the main model for imputation is. You write: "We assumed that missing DOS occur at random with respect to symptom onset date, and that reporting delays conditional on DOR can be modeled over time t and location*

*i as a negative binomial distribution with mean parameter \mu_{i,t} and dispersion parameter \theta_i estimated using maximum likelihood [9]."Based on the code from github, it looks like you utilized a model log(\mu_{i,t}) = \beta_{0,i} + \beta_{1,i}*t i.e., you assumed a linear time-trend for modeling the (log)-expectation of the reporting delay. This seems to be a rather unflexible model, but might be flexible enough for your data. However, I am not sure if you really used this model as you later say with respect to the sensitivity analyses: "mean parameter mu_{i,t} and dispersion parameter theta_i,t [were] estimated from the distribution of observed delays pooled from t − tau to t (weaker assumption than the main approach with \tau=7)". I am somewhat confused what the final "main approach" is, please clarify this and specify the model using a clear description or formula!*

Thank you. We have now reassessed and clarified the model, with details in the Supplementary information, Section S1. In general, for the main analysis for each day t and location i we now use the true observations on day t +/-1 to estimate \mu_{i,t} and \theta_{i,t} (instead of theta{i}). This represents a more flexible version of the model where we estimate theta by day instead of using a single value for the whole period, under the assumption that dispersion varies over time.

***“Section S1. Additional information on model specification***

*Imputation model*

*For our imputation step, we first assume that missing DOS occurs at random with respect to symptom onset date. Further, we assume that both observed and missing reporting delays* $Y$ *conditional on DOR can be approximated over time*$t$*and location* $i$*as a negative binomial distribution with mean parameter* $\mu_{i,t}$ *and dispersion parameter* $\theta_{i, t}$*:*

$Y_{i,t}$*~*$NB(\mu_{i,t},\theta_{i, t})$	*eq 1.*

*where* $\mu_{i,t}$*and* $\theta_{i, t}$*can be modeled as:*

$\mu_{i,t}$*~*$Normal$*(*$\mu_{i,t},\sigma_{i,t}$*)	eq 2.*

$\theta_{i, t}$*~*$Normal$*(*$\mu_{i,t},\sigma_{i,t}$*)	eq 3.*

*For each day t and location i, we estimate* $\hat{\mu}_{i,t}$ *and* $\hat{\theta}_{i,t}$ *estimate by maximum likelihood estimation* [*[9]*](https://paperpile.com/c/k3VeiC/4Xck9) *using the log-link on eq 1. and the observations on day* $t+/-1$*. We also estimate the standard error of the mean for each parameter. Using observations on days* $t+/-1$ *instead of only* $i$ *allows us to increase size under the hypothesis that report delays do not change significantly at a daily scale. We then generate random samples using the model in eq 1- 3 with the mean of the parameters in eq. 2 and 3 set to* $\hat{\mu}_{i,t}$ *and estimate* $\hat{\theta}_{i,t}$ *and using their estimated standard errors as standard deviations. We resampled 100 times to generate 100 time series of cases with complete DOS-DOR for each day and region* $i$*. The sum of the observed and imputed cases became the total cases used for nowcasting. Because negative binomial estimates can be biased in small samples* [*[31,32]*](https://paperpile.com/c/k3VeiC/dWjk+wbeS)*, we simulated the delays using eq 1-3 with* $\mu_{i,t}$ *replaced by* $\hat{\mu}_{i}$ *estimated from all past observations and with* $\theta_{i,}$ *set to 1 when the number of observations was n <50 . This approach is consistent with the moderate-to-high dispersion assumption (i.e. small* $\theta$ *in eq. 1) and ensures that the probability of delay*$d$ *is always greater than the probability for delay* $d+1$*. Fixing* $\theta_{i, t}$ *to 0.5 or 2, or setting it to 1 when n<100, resulted in similar estimates. Allowing* $\theta_{i, t}$*to be parameterized from the observations (when the number of individuals with complete DOS-DOR on* $t+/-1$ *was smaller than 50) instead of fixing it led to a slight underestimation relative to the nowcast, likely because the true delay distribution is commonly zero-inflated though still overdispersed when a high proportion of cases are missing, and low numbers are reported. Further sensitivity analyses need to be developed to address this limitation more systematically.”*

We also have reworded the corresponding sensitivity to be consistent with the changes in the methods:

*“ (…) we repeated the analyses after masking DOS (hiding original data as missing values) in a random 10% and a random 40% of reported cases. Second, we used two alternative parameterizations for the reporting delay (after masking DOS in a random 40% of cases): a) a single mean parameter* $\mu$*over days and region (stronger assumption than the main approach) b) mean parameter* $\mu_{i,t}$ *and dispersion parameter* $\theta_{i,t}$ *estimated from the distribution of observed delays pooled from* $t-\tau$ *to* $t$*, using* $\tau$*=7 (stronger assumption than the main approach but weaker than a).”*

*And the sensitivity section S2:*

*“However, when a high proportion of cases are censored, the imputation model smooths the epidemic curve and reduces its initial slopes and global maxima. The smoothing was greater when using stronger assumptions (less flexible parameterization, see methods). The Mean Absolute Percentage Errors (MAPE) did not substantially differ for the main model vs the alternatives with stronger assumptions were 13.1%, 14.6% and 14.2% respectively for Murcia, and 11.4%, 12.2%, 11.2% for Madrid.”*

*5) For incorporating the imputation uncertainty into the nowcast you write that you "resampled 100 times to generate 100 time series of cases with complete DOS-DOR for each region i, allowing \mu_{i,t} and \theta_i to vary randomly under a normal distribution with the standard deviation \sigma set to the sampling error." I am not sure what you mean by "sampling error". I assume that you sample from the negative binomial distribution with parameters coming from draws of the normal distribution with parameters \hat{mu} and \hat{SE} of the corresponding parameters from the imputation model? It would then probably be better to replace "Sampling error" by "standard error" and also specify the expectation of the normal distribution you are sampling from.*

Thank you. We have changed the text as suggested. Also, see response to previous comment with regards to the model specification.

*Additional general comments: 6) What do the vertical lines in Figure 3BD indicate? They do not seem to correspond to the time-points when R(t) crosses 1.*

Thank you. That was an error that has been corrected.

*7) Furthermore, you might consider plotting R(t) on a log-scale as this better describes the multiplicative interpretation of the reproductive number (and increases readability of your figures with very high values from the raw case counts).*

Thank you. We have now used the log-scale for Figure 3. However, we have kept the continuous scale in the main Figure 1 because we believe readers are more used to that format and high values are not a problem for those estimates.

*In the discussion, you mention that "Our findings showed that a country-wide lockdown control led to a substantial decline in cases shortly thereafter, around March 14-20 in Madrid and around March 16-21 in Murcia." I think that such causal statements are not covered by the presented analyses and it would be better to speak of a temporal association. I would also refrain from comparing the nowcasting results and the curve of case counts with respect to their consistency with the date of the country-wide lockdown in the Results section.*

We have now removed the comparison from results and changed the statement as suggested:

*“Our findings showed that a country-wide lockdown control was followed by a substantial decline in cases shortly thereafter, around March 14-20 in Madrid and around March 17-23 in Murcia.”*

*The description of the first-order Random walk in Section S1 seems to include a typo. Expectation of the normal distribution should probably be \alpha_{t-1}*

Thank you, the typo has been corrected.
